# Risk scores for predicting small for gestational age infants in Japan: The TMM birthree cohort study

**DOI:** 10.1038/s41598-022-12892-0

**Published:** 2022-05-26

**Authors:** Noriyuki Iwama, Taku Obara, Mami Ishikuro, Keiko Murakami, Fumihiko Ueno, Aoi Noda, Tomomi Onuma, Fumiko Matsuzaki, Tetsuro Hoshiai, Masatoshi Saito, Hirohito Metoki, Junichi Sugawara, Nobuo Yaegashi, Shinichi Kuriyama

**Affiliations:** 1 Department of Obstetrics and Gynecology, Tohoku University Hospital, 1-1, Seiryomachi, Sendai, Miyagi 980-8574 Japan; 2 Division of Molecular Epidemiology, Department of Preventive Medicine and Epidemiology, Tohoku Medical Megabank Organization, Tohoku University, Sendai, Japan; 3 Division of Molecular Epidemiology, Graduate School of Medicine, Tohoku University, Sendai, Japan; 4 Department of Pharmaceutical Sciences, Tohoku University Hospital, Sendai, Miyagi Japan; 5 Department of Maternal and Fetal Therapeutics, Tohoku University Graduate School of Medicine, Sendai, Miyagi Japan; 6 Tohoku Medical Megabank Organization, Tohoku University, Sendai, Miyagi Japan; 7 Division of Public Health, Hygiene and Epidemiology, Tohoku Medical Pharmaceutical University, Sendai, Miyagi Japan; 8 Environment and Genome Research Center, Tohoku University Graduate School of Medicine, Sendai, Miyagi Japan; 9 Department of Obstetrics and Gynecology, Tohoku University Graduate School of Medicine, Sendai, Miyagi Japan; 10 International Research Institute of Disaster Science, Tohoku University, Sendai, Miyagi Japan

**Keywords:** Epidemiology, Risk factors

## Abstract

This study aimed to construct a prediction model for small-for-gestational-age (SGA) infants in Japan by creating a risk score during pregnancy. A total of 17,073 subjects were included in the Tohoku Medical Megabank Project Birth and Three-Generation Cohort Study, a prospective cohort study. A multiple logistic regression model was used to construct risk scores during early and mid-gestational periods (11–17 and 18–21 weeks of gestation, respectively). The risk score during early gestation comprised the maternal age, height, body mass index (BMI) during early gestation, parity, assisted reproductive technology (ART) with frozen-thawed embryo transfer (FET), smoking status, blood pressure (BP) during early gestation, and maternal birth weight. The risk score during mid-gestation also consisted of the maternal age, height, BMI during mid-gestation, weight gain, parity, ART with FET, smoking status, BP level during mid-gestation, maternal birth weight, and estimated fetal weight during mid-gestation. The C-statistics of the risk scores during early- and mid-gestation were 0.658 (95% confidence interval [CI]: 0.642–0.675) and 0.725 (95% CI: 0.710–0.740), respectively. In conclusion, the predictive ability of the risk scores during mid-gestation for SGA infants was acceptable and better than that of the risk score during early gestation.

## Introduction

Small for gestational age (SGA) infants, a common surrogate of fetal growth restriction (FGR), is a risk factor for adverse perinatal events^1–4^. Infants born SGA or with FGR are at risk for stillbirth, non-reassuring fetal status, perinatal asphyxia, neonatal death, and neurological developmental delay^1–4^. Infants who are diagnosed as SGA after birth have a higher risk of developing severe adverse complications than those who are diagnosed as SGA before birth^5,6^. By judgement of the delivery management method following fetal surveillance using ultrasonography and cardiotocography, the risk of fetal and/or adverse neonatal complications, including severe fetal distress and fetal/infant death, may decrease ^5,6^. Therefore, detection of pregnant women at a high risk of delivering SGA infants is important for careful fetal surveillance^7^. Healthcare providers evaluate infant birth weight as the estimated fetal weight (EFW) during the fetal period using ultrasonography (a simple and accepted method) at the prenatal checkup; however, not all SGA infants are detected due to overestimation of the EFW^8,9^. Therefore, using a prediction model for the early identification of pregnant women at a high risk of delivering SGA infants, and subsequent careful prenatal checkups and fetal surveillance before delivery, may lead to preferable outcomes.

Thus far, previous studies in several countries have reported prediction models based on the clinical risk factors, ultrasound findings, and biomarkers of SGA infants^10–14^. Furthermore, several studies in Japan have also reported certain factors associated with infant birth weight; however, racial differences exist in the maternal and neonatal characteristics, including the maternal physique and infant birth weight^15–22^. Pre-pregnancy body mass index (BMI) and recommendations for weight gain during pregnancy are different across countries^20,21^. A possible reason for the difference in mean infant birth weight between Japanese and White people is differences in maternal physique and weight gain during pregnancy^23^.

Due to lack of previous studies on pregnant Japanese women reporting a prediction model for SGA infants, this study aimed to construct a prediction model for SGA infants in Japan by creating a risk score during pregnancy.

## Results

### Maternal and neonatal characteristics of the study subjects

**Figure 1:**
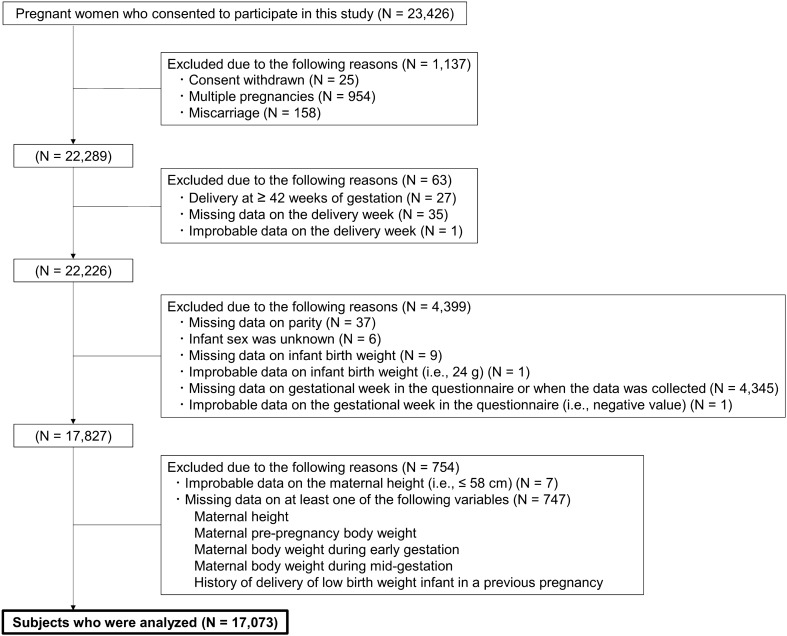
Flow chart of the study participants.

Figure 1 presents a flow chart of the study subjects. Of the 23,425 pregnant women who consented to participate in the Tohoku Medical Megabank Project (TMM) Birth and Three-Generation Cohort Study (TMM BirThree Cohort Study), participants were excluded due to the following reasons: withdrawn consent (N = 25); multiple pregnancies (N = 954); miscarriages (N = 158); delivery week at ≥ 42 weeks of gestation (N = 27); missing data (N = 35) and improbable data (N = 1) on the delivery week; missing data on parity (N = 37); unknown infant sex (N = 6); missing data on infant birth weight (N = 9); improbable data on infant birth weight (i.e., 24 g) (N = 1); missing data on the gestational week in the questionnaire or when the data was collected (N = 4,345); and improbable data on the gestational week in the questionnaire (N = 1). Furthermore, seven women with improbable data on the maternal height were excluded followed by 747 women who were excluded due to missing data on at least one of the following variables: maternal height, pre-pregnancy body weight (BW), BW during early gestation, BW during mid-gestation, and history of delivery of low birth weight (LBW) infants in a previous pregnancy. Finally, 17,073 women remained and were subsequently analyzed. Women with “No answer” on questions regarding the maternal birth weight and smoking status were not excluded considering the clinical use of risk scores to predict SGA infants.

Table 1 shows the maternal and neonatal characteristics of the study subjects. The number of SGA infants was 1,126 (6.6%).

**Table 1:** Maternal and neonatal characteristics of the study subjects. Continuous variables and categorical variables are expressed as mean ± SD and number (%), respectively. Abbreviations: AIH, artificial insemination by the husband; APS, antiphospholipid syndrome; ART, assisted reproductive technology; BMI, body mass index; BW, body weight; DBP, diastolic blood pressure; EFW, estimated fetal weight; ET, embryo transfer; FET, frozen-thawed ET; HDP, hypertensive disorders of pregnancy; ICSI, intracytoplasmic sperm injection; IQR, interquartile range; IVF, in vitro fertilization; LBW, low birth weight; SBP, systolic blood pressure; SD, standard deviation; SGA, small for gestational age; SLE, systemic lupus erythematosus.

Variables	Values of the study subjects (N = 17,073)	The numbers and proportion of SGA infants in each category, cases/number (%)
**Maternal characteristics**		
**Gestational week at prenatal checkup during early gestation, median (IQR)**	12.4 (11.7–13.1)	–
**Gestational week at prenatal checkup during mid-gestation, median (IQR)**	20.1 (19.4–20.6)	–
**Age during early gestation, years**	31.8 ± 5.0	–
< 25 years, n (%)	1,496 (8.8)	92/1,496 (6.2)
25–29.9 years, n (%)	4,685 (27.4)	308/4,685 (6.6)
30–34.9 years, n (%)	6,271 (36.7)	403/6,271 (6.4)
≥ 35 years, n (%)	4,621 (27.1)	323/4,621 (7.0)
**Age during mid-gestation, years**	31.9 ± 5.0	–
< 25 years, n (%)	1,412 (8.3)	86/1,412 (6.1)
25–29.9 years, n (%)	4,609 (27.0)	302/4,609 (6.6)
30–34.9 years, n (%)	6,275 (36.8)	404/6,275 (6.4)
≥ 35 years, n (%)	4,777 (28.0)	334/4,777 (7.0)
**Height, cm**	158.3 ± 5.4	-
Quartile 1 (< 155 cm), n (%)	4,122 (24.1)	374/4,122 (9.1)
Quartile 2 (155–158.9 cm), n (%)	4,986 (29.2)	352/4,986 (7.1)
Quartile 3 (159–161.9 cm), n (%)	3,345 (19.6)	205/3,345 (6.1)
Quartile 4 (≥ 162 cm), n (%)	4,620 (27.1)	195/4,620 (4.2)
**Pre-pregnancy BW, kg**	54.3 ± 9.5	–
**Initial BW during early gestation, kg**	54.7 ± 9.4	–
**Initial BW during mid-gestation, kg**	57.2 ± 9.2	–
**Pre-pregnancy BMI, kg/m**^**2**^	21.6 ± 3.5	–
Quartile 1 (< 19.3 kg/m^2^), n (%)	4,237 (24.8)	379/4,237 (9.0)
Quartile 2 (≥ 19.3 and < 20.8 kg/m^2^), n (%)	4,063 (23.8)	268/4,063 (6.6)
Quartile 3 (≥ 20.8 and < 23.0 kg/m^2^), n (%)	4,462 (26.1)	276/4,462 (6.2)
Quartile 4 (≥ 23.0 kg/m^2^), n (%)	4,311 (25.3)	203/4,311 (4.7)
**Initial BMI during early gestation, kg/m**^**2**^	21.8 ± 3.5	-
Quartile 1 (< 19.5 kg/m^2^), n (%)	4,263 (25.0)	381/4,263 (8.9)
Quartile 2 (≥ 19.5 and < 21.1 kg/m^2^), n (%)	4,217 (24.7)	292/4,217 (6.9)
Quartile 3 (≥ 21.1 and < 23.2 kg/m^2^), n (%)	4,225 (24.8)	242/4,225 (5.7)
Quartile 4 (≥ 23.2 kg/m^2^), n (%)	4,368 (25.6)	211/4,368 (4.8)
**Initial BMI during mid-gestation, kg/m**^**2**^	22.8 ± 3.4	-
Quartile 1 (< 20.5 kg/m^2^), n (%)	4,101 (24.0)	387/4,101 (9.4)
Quartile 2 (≥ 20.5 and < 22.1 kg/m^2^), n (%)	4,263 (25.0)	303/4,263 (7.1)
Quartile 3 (≥ 22.1 and < 24.2 kg/m^2^), n (%)	4,377 (25.6)	246/4,377 (5.6)
Quartile 4 (≥ 24.2 kg/m^y^), n (%)	4,332 (25.4)	190/4,332 (4.4)
**Weight gain between pre-pregnancy and early gestation** **(Initial BW during early gestation − pre-pregnancy BW), kg**	0.5 ± 2.5	–
Quartile 1 (< -0.6 kg), n (%)	4,370 (25.6)	288/4,370 (6.6)
Quartile 2 (≥ -0.6 and < 0.6 kg), n (%)	4,257 (24.9)	289/4,257 (6.8)
Quartile 3 (≥ 0.6 and < 1.7 kg), n (%)	4,086 (23.9)	268/4,086 (6.6)
Quartile 4 (≥ 1.7 kg), n (%)	4,360 (25.5)	281/4,360 (6.4)
**Weight gain between early and mid-gestation** **(Initial BW during mid-gestation − Initial BW during early gestation), kg**	2.4 ± 1.8	–
Quartile 1 (< 1.5 kg), n (%)	4,098 (24.0)	361/4,098 (8.8)
Quartile 2 (≥ 1.5 and < 2.4 kg), n (%)	4,527 (26.5)	346/4,527 (7.6)
Quartile 3 (≥ 2.4 and < 3.4 kg), n (%)	4,333 (25.4)	240/4,333 (5.5)
Quartile 4 (≥ 3.4 kg), n (%)	4,115 (24.1)	179/4,115 (4.4)
**Parity, n (%)**		
Primipara	8,073 (47.3)	529/8,073 (6.6)
Multipara without HDP or delivery of LBW infants in a previous pregnancy	7,676 (45.0)	427/7,676 (5.6)
Multipara with HDP and/or delivery of LBW infants in a previous pregnancy	1,324 (7.8)	170/1,324 (12.8)
**Conception method, n (%)**		
Natural pregnancy	15,883 (93.0)	1,059/15,883 (6.7)
Non-ART (ovulation induction or AIH)	388 (2.3)	27/388 (7.0)
ART (conventional IVF or ICSI) with fresh ET	80 (0.5)	5/80 (6.3)
ART (conventional IVF or ICSI) with FET	489 (2.9)	18/489 (3.7)
ART (conventional IVF or ICSI) without information on the method of ET	203 (1.2)	14/203 (6.9)
Others	30 (0.2)	3/30 (10.0)
**Maternal birth weight, n (%)**		
< 2,500 g	783 (4.6)	91/783 (11.6)
2,500–2,999 g	3,160 (18.5)	282/3,160 (8.9)
3,000–3,499 g	4,925 (28.9)	245/4,925 (5.0)
≥ 3,500 g	1,298 (7.6)	31/1,298 (2.4)
Unknown or No answer	6,907 (40.5)	477/6,907 (6.9)
**Medical history, n (%)**		
Diabetes mellitus		
No	17,010 (99.6)	1,122/17,010 (6.6)
Yes	63 (0.4)	4/63 (6.4)
SLE and/or APS		
No	17,042 (99.8)	1,122/17,042 (6.6)
Yes	31 (0.2)	4/31 (12.9)
Chronic kidney disease		
No	17,022 (99.7)	1,123/17,022 (6.6)
Yes	51 (0.3)	3/51 (5.9)
Hyperthyroidism		
No	16,683 (98.9)	1,115/16,683 (6.6)
Yes	190 (1.1)	11/190 (5.8)
Hypothyroidism		
No	16,836 (98.6)	1,108/16,836 (6.6)
Yes	237 (1.4)	18/237 (7.6)
**Smoking status, n (%)**		
Never	10,202 (59.8)	683/10,202 (6.7)
Quit smoking before conception	4,003 (23.5)	248/4,003 (6.2)
Quit smoking after conception	2,388 (14.0)	137/2,388 (5.7)
Continue smoking during pregnancy	412 (2.4)	49/412 (11.9)
No answer	68 (0.4)	9/68 (13.2)
**Alcohol drinking, n (%)**		
Constitutionally never drinker	990 (5.8)	69/990 (7.0)
Almost never drinking or Quit drinking	12,738 (74.6)	836/12,738 (6.6)
Continue drinking during pregnancy	3,290 (19.3)	218/3,290 (6.6)
No answer	55 (0.3)	3/55 (5.5)
**Initial clinic blood pressure level during early gestation**		
Normal blood pressure (SBP is < 120 mmHg and DBP is < 80 mmHg)	12,726 (74.5)	854/12,726 (6.7)
High normal blood pressure (SBP is 120–129 mmHg and DBP is < 80 mmHg)	2,520 (14.8)	145/2,520 (5.8)
Elevated blood pressure (SBP is 130–139 mmHg and/or DBP is 80–89 mmHg)	1,423 (8.3)	89/1,423 (6.3)
Grade 1 or higher hypertension (SBP is ≥ 140 mmHg and/or DBP is ≥ 90 mmHg)	404 (2.4)	38/404 (9.4)
**Initial clinic blood pressure level during mid-gestation**		
Normal blood pressure (SBP is < 120 mmHg and DBP is < 80 mmHg)	13,465 (78.9)	892/13,465 (6.6)
High normal blood pressure (SBP is 120–129 mmHg and DBP is < 80 mmHg)	2,436 (14.3)	147/2,436 (6.0)
Elevated blood pressure (SBP is 130–139 mmHg and/or DBP is 80–89 mmHg)	926 (5.4)	61/926 (6.6)
Grade 1 or higher hypertension (SBP is ≥ 140 mmHg and/or DBP is ≥ 90 mmHg)	246 (1.4)	26/246 (10.6)
**Initial SD value of the EFW during mid-gestation, SD**	0.3 ± 0.9	–
≥ + 1.5 SD	1,441 (8.4)	35/1,441 (2.4)
≥ + 0.5 SD and < + 1.5 SD	5,414 (31.7)	208/5,414 (3.8)
> -0.5 SD and < + 0.5 SD	7,821 (45.8)	524/7,821 (6.7)
> -1.5 SD and ≤ -0.5 SD	2,228 (13.1)	320/2,228 (14.4)
≤ -1.5 SD	169 (1.0)	39/169 (23.1)
**Obstetric complications, n (%)**		
HDP	846 (5.0)	124/846 (14.7)
Gestational diabetes mellitus	438 (2.6)	28/438 (6.4)
Placental abruption	34 (0.2)	5/34 (14.7)
Placenta previa	102 (0.6)	3/102 (2.9)
Low-lying placenta	67 (0.4)	1/67 (1.5)
Intrauterine fetal death	8 (0.1)	4/8 (50.0)
Stillbirth	12 (0.1)	6/12 (50.0)
**Neonatal characteristics**		
**Infant sex (male/female), n (%)/n (%)**	8,837 (51.8)/ 8,236 (48.2)	583/8,837 (6.6) / 543/8,236 (6.6)
**Major congenital anomalies**	328 (1.9)	35/328 (10.7)
**Chromosomal abnormality**		
Trisomy 21	19 (0.1)	2/19 (10.5)
Trisomy 18	5 (0.03)	4/5 (80.0)
Trisomy 13	2 (0.01)	1/2 (50.0)
**Skeletal dysplasia**		
Thanatophoric dysplasia	3 (0.01)	0/3 (0.0)
Achondrogenesis	1 (0.01)	0/1 (0.0)
Achondroplasia	1 (0.01)	0/1 (0.0)
Osteogenesis imperfecta	0 (0.0)	–
**Other major congenital anomalies**	302 (1.8)	29/302 (9.6)
**Gestational age at delivery, weeks**	39.2 ± 1.7	38.7 ± 2.7
Preterm birth (Delivery at less than 37 weeks of gestation)	880 (5.2)	125/880 (14.2)
Preterm birth (Delivery at less than 34 weeks of gestation)	196 (1.2)	55/196 (28.1)
Preterm birth (Delivery at less than 32 weeks of gestation)	131 (0.8)	36/131 (27.5)
**Infant birth weight**		
Grams	3,031 ± 430	2,349 ± 431 in SGA infants
SD value	0.2 ± 1.0	-1.8 ± 0.6 in SGA infants
LBW infants (birth weight < 2,500 g), n (%)	1,395 (8.2)	638/1,395 (45.7)
SGA infants (birth weight < 10^th^ percentile), n (%)	1,126 (6.6)	–
Preterm SGA infants (Birth weight < 10th percentile and preterm birth from 22 to < 37 weeks of gestation)	125 (0.7)	–
Term SGA infants (Birth weight < 10th percentile and birth from 37 to < 42 weeks of gestation)	1,001 (5.9)	–

### Risk scores during early and mid-gestation for predicting SGA infants

Supplementary Table S1 and the supplementary information present the results of the univariate logistic regression model. Supplementary Tables S2, S3, and S4 show the adjusted odds ratios, regression coefficients, and integer scores during the early- and mid-gestation periods based on the multiple logistic regression model. The selected explanatory variables in all risk scores were maternal height, parity, assisted reproductive technology (ART; conventional in vitro fertilization or intracytoplasmic sperm injection) with frozen-thawed embryo transfer (FET), continued smoking during pregnancy, and maternal birth weight. The maternal age during early gestation, maternal BMI during early pregnancy, and hypertension (grade 1 or higher) during early gestation were also selected while creating the risk scores during early gestation. When the risk score during mid-gestation was constructed, the maternal age during mid-gestation, maternal BMI during mid-gestation, weight gain between the initial body weight (BW) during early gestation and the BW during mid-gestation, and hypertension (grade 1 or higher) during mid-gestation were selected for model 1. For model 2 during mid-gestation, the initial standard deviation (SD) value of the EFW during mid-gestation was also selected in addition to the parameters selected for model 1.

### Model performance and calibration plot of the risk scores for predicting SGA infants

**Figure 2:**
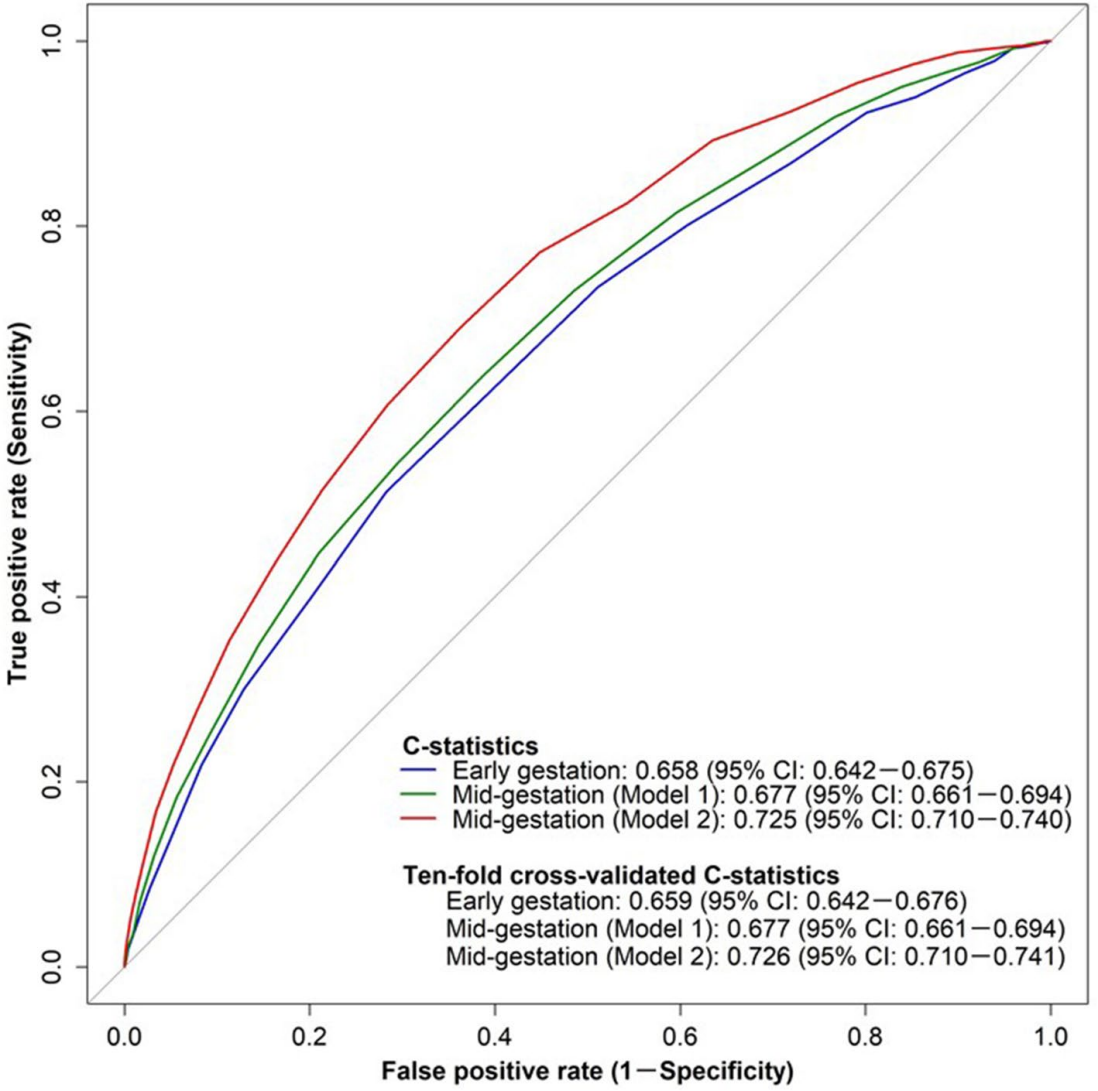
Receiver operating characteristics curve of each risk score for predicting SGA infants Abbreviations: CI, confidence interval; EFW, estimated fetal weight; SGA, small for gestational age.

The discrimination performance of each risk score is shown in Fig. 2. The risk score during early gestation showed a poor discrimination performance. Models 1 and 2 (i.e., risk scores during mid-gestation) showed poor and acceptable discrimination performances, respectively. The C-statistics and ten-fold cross-validated C-statistics of model 2 during mid-gestation were 0.725 (95% confidence interval [CI]: 0.710–0.740) and 0.726 (95% CI: 0.710–0.741), respectively. The results of the sensitivity analysis performed using multiple imputation by a chained equation are described in the supplementary information.

**Figure 3:**
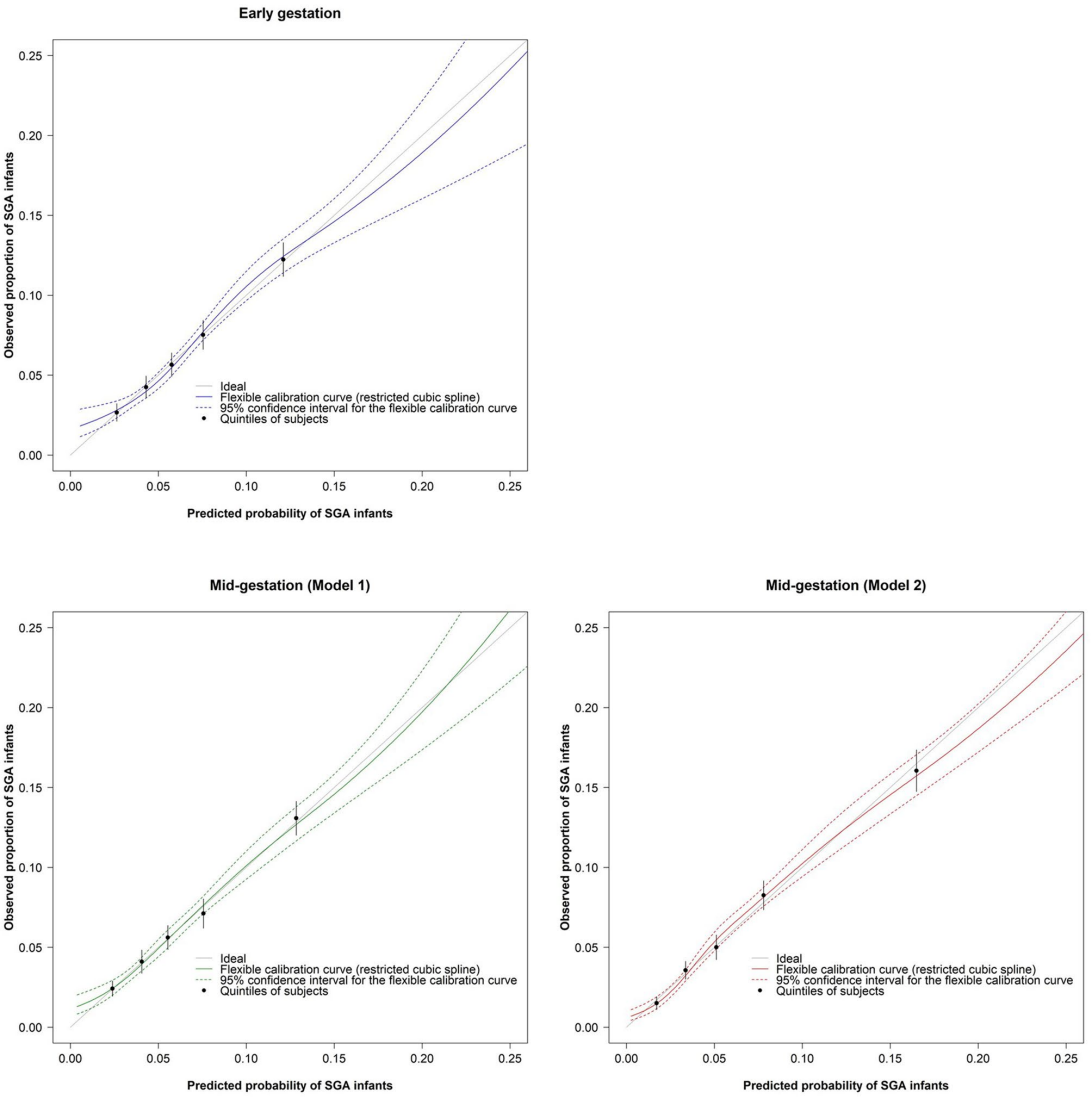
Calibration plot of each risk score for predicting SGA infants. “Ideal” indicates the line of perfect calibration. Each black circle and vertical line show the mean and 95% confidence interval of the proportion of SGA infants in each quintile of the risk score, respectively.

**Figure 4:**
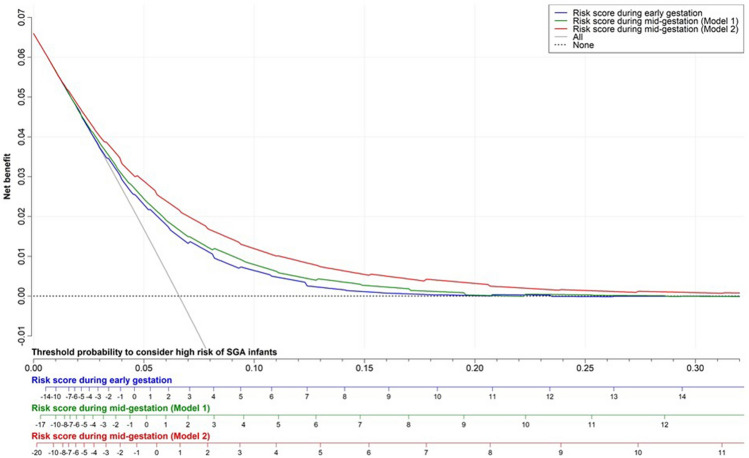
Decision curve analysis of the risk scores for predicting SGA infants. “All” (solid grey line) indicates that all subjects are considered to be at a high risk of delivering SGA infants. “None” (dashed black line) indicates that no subjects are considered to be at a high risk of delivering SGA infants.

Table 2 shows the observed proportion of the SGA infants and the predicted probability of the SGA infants according to the quintiles of each risk score. As shown in Fig. 3, the possibility of miscalibration was low, because the calibration curve in each risk score was close to the diagonal line (i.e., the line of perfect calibration). Figure 4 shows the result of the decision curve analysis. The risk score during early gestation, model 1, and model 2 during mid-gestation had a higher net benefit (NB) than that of either all or no subjects considered to be at a high risk of delivering SGA infants when the threshold probabilities were 0.029–0.183 (risk score = − 3 to 10); 0.023–0.195 (risk score = − 5 to 9), and 0.019–0.312 (risk score = − 6 to 11), respectively.

**Table 2:** Predicted probability and observed proportion of SGA infants according to the quintiles of each risk score. The risk score during early gestation comprised the maternal age, height, BMI during early gestation, parity, ART with FET, smoking status, BP during early gestation, and maternal birth weight. The risk score during mid-gestation (model 1) consisted of the maternal age, height, BMI during mid-gestation, weight gain, parity, ART with FET, smoking status, BP level during mid-gestation, and maternal birth weight. The risk score during mid-gestation (model 2) also consisted of the maternal age, height, BMI during mid-gestation, weight gain, parity, ART with FET, smoking status, BP level during mid-gestation, maternal birth weight, and estimated fetal weight during mid-gestation.

Total risk score	Predicted probability of SGA infants (95% CI), %	Observed proportion of SGA infants (Cases/total), %
**Early gestation**		
Quintile 1 (≤ -2)	2.6 (2.1–3.2)	2.7 (87/3,262)
Quintile 2 (-1 to 0)	4.3 (3.6–5.0)	4.3 (137/3,222)
Quintile 3 (1 to 2)	5.8 (4.9–6.4)	5.7 (208/3,679)
Quintile 4 (3 to 4)	7.5 (6.6–8.4)	7.5 (242/3,217)
Quintile 5 (≥ 5, High risk)	12.1 (11.2–13.3)	12.2 (452/3,693)
Predicted probability of SGA infants based on the risk score	exp(logit)/(1 + exp[logit]) where logit = -3.0394 + 0.1545 × (risk score)	–
**Model 1 during mid-gestation**		
Quintile 1 (≤ -3)	2.4 (1.9–2.9)	2.4 (92/3,808)
Quintile 2 (-2 to -1)	4.1 (3.4–4.8)	4.1 (116/2,831)
Quintile 3 (0 to 1)	5.5 (4.9–6.4)	5.6 (200/3,564)
Quintile 4 (2 to 3)	7.6 (6.2–8.0)	7.1 (215/3,023)
Quintile 5 (≥ 4, High risk)	12.9 (12.0–14.1)	13.1 (503/3,847)
Predicted probability of SGA infants based on the risk score	exp(logit)/(1 + exp[logit]) where logit = -2.9199 + 0.1671 × (risk score)	–
**Model 2 during mid-gestation** **(Model 1 + EFW)**		
Quintile 1 (≤ -5)	1.7 (1.1–1.9)	1.5 (51/3,199)
Quintile 2 (-4 to -2)	3.4 (3.0–4.1)	3.6 (146/4,099)
Quintile 3 (-1 to 0)	5.1 (4.2–5.8)	5.0 (151/3,017)
Quintile 4 (1 to 3)	7.8 (7.4–9.2)	8.3 (292/3,538)
Quintile 5 (≥ 4, High risk)	16.5 (14.7–17.4)	16.1 (486/3,028)
Predicted probability of SGA infants based on the risk score	exp(logit)/(1 + exp[logit]) where logit = -2.8282 + 0.1853 × (risk score)	–

Abbreviations: ART; assisted reproductive technology, BMI; body mass index; BP, blood pressure; CI, confidence interval; exp, exponential; EFW, estimated fetal weight; FET, frozen-thawed embryo transfer; SGA, small for gestational age.

### Comparison of model performance between different risk scores for predicting SGA infants

Supplementary Table S5 shows the results of a comparison of the model performance between different risk scores for the prediction of SGA infants. In terms of discrimination and reclassification, model 2 showed a better performance for predicting SGA infants during mid-gestation as compared to the risk score during early gestation or model 1 during mid-gestation. As shown in Fig. 4, the NB in model 2 during mid-gestation was higher than that in the risk score during early gestation and model 1 during mid-gestation.

### Discrimination and clinical utility based on different cut-off values of the risk scores for prediction of SGA infants

Table 3 shows the discrimination performance and the NB based on the lowest risk score of quintile 5 as the cut-off value in each risk score. In model 2 during mid-gestation, the lowest risk score of quintile 5 as the cut-off value (i.e., risk score = 4) was equivalent to a threshold probability of 0.110 and had an NB of 0.011. Supplementary Table S6 also shows the discrimination performance and NB when each risk score that was closest to several threshold probabilities was set as the cut-off value. Supplementary Table S7 illustrates the discrimination performance and NB for each of the risk score that had the maximum Youden index that was set as the cut-off value.

**Table 3:** Discrimination and the NB based on the lowest risk score of quintile 5 as the cut-off value in each risk score.

Cut-off (Minimum risk score of quintile 5)	TPR (Sensitivity) (95% CI)	Specificity (95% CI)	PPV (95% CI)	NPV (95% CI)	Positive LR (95% CI)	Negative LR (95% CI)	NB
**Early gestation**							
Risk score = 5 (Predicted probability of SGA infants = 0.094)	0.401 (0.373–0.430)	0.797 (0.791–0.803)	0.122 (0.112–0.133)	0.950 (0.946–0.953)	1.98 (1.83–2.14)	0.75 (0.72–0.79)	0.007
**Mid-gestation**							
Model 1: Risk score = 4 (Predicted probability of SGA infants = 0.095)	0.447 (0.418–0.476)	0.790 (0.784–0.797)	0.131 (0.120–0.141)	0.953 (0.949–0.957)	2.13 (1.98–2.29)	0.70 (0.66–0.74)	0.008
Model 2: Risk score = 4 (Predicted probability of SGA infants = 0.110)	0.432 (0.403–0.461)	0.841 (0.835–0.846)	0.161 (0.147–0.174)	0.954 (0.951–0.958)	2.71 (2.51–2.92)	0.68 (0.64–0.71)	0.011

Abbreviations: CI, confidence interval; LR, likelihood ratio; NB, net benefit; NPV, negative predictive value; PPV, positive predictive value; SGA, small for gestational age; TPR, true positive rate.

### Model performance, calibration, and clinical utility of each risk score for predicting preterm and term SGA infants (Results of an additional analysis)

The results of the model performance, calibration, and clinical utility of each risk score for predicting preterm and term SGA infants, an additional analysis performed in the present study, are described in the Supplementary Information.

## Discussion

This is the first study in Japan to construct a prediction model for SGA infants based on risk scores. Since the predictive ability of the risk score including the EFW during mid-gestation was acceptable, it can be incorporated into prenatal checkup protocols for the early detection of pregnant women at a high risk of delivering SGA infants in Japan.

The predictive ability of the risk scores for SGA infants during early gestation was poor. However, by using a risk score, healthcare providers and pregnant women can collectively identify certain risk factors to improve outcomes, for instance, the smoking status during pregnancy, which can be modified through smoking cessation^24,25^. Maternal smoking during pregnancy is associated with higher resistance within the umbilical artery flow^26^. Decrease in endothelial nitric oxide synthase (eNOS) activity in fetal umbilical and chorionic vessels, caused by maternal smoking, may be a possible mechanism^27^. In addition, eNOS activity in pregnant women who quit smoking during pregnancy was higher than in those who smoked during pregnancy^27^. Therefore, smoking cessation may lead to a decrease in risk of SGA infants by increasing eNOS activity in fetal umbilical and chorionic vessels. Probably because model 1 during mid-gestation included the maternal weight gain and was closer to the time of delivery than the risk score during early gestation, it had a higher ability to predict SGA infants. By using a risk score during mid-gestation, healthcare providers can recognize maternal weight gain, which may lead to possible nutrition counseling according to the maternal physique. The predictive ability of the risk score including the EFW during mid-gestation for SGA infants in this study was similar to that of other prediction models^10,14^. McCowan et al. reported that the C-statistic of the prediction model for SGA infants, created using clinical data and ultrasound variables collected at 15 and 20 weeks of gestation, respectively, was 0.73^10^. Erkamp et al. also reported that the C-statistic of the prediction model for SGA infants using maternal characteristics and the EFW during the second trimester was 0.72 (95% CI: 0.70–0.74)^14^.

Considering maternal complications, SGA (as a surrogate of FGR) requires careful surveillance with modalities such as the EFW, fetal doppler velocimetry, and cardiotocography at tertiary institutions^7^. If pregnant women are considered at a high-risk of delivering SGA infants by the prediction model, a detailed fetal ultrasonography will be needed. Fetal ultrasonography includes confirmation of congenital morphological abnormality, measuring maternal uterine artery pulsatility index for evaluation of placental dysfunction, close follow-up of EFW and fetal abdominal circumference for evaluation of fetal growth velocity. If fetal growth deteriorates, evaluation of fetal doppler velocimetry, including umbilical artery pulsatility index, middle cerebral artery pulsatility index, and flow of ductus venosus in combination with cardiotocography or biophysical profile scoring will be needed. In Japan, pregnant women are commonly managed at midwife homes or at primary, secondary, and tertiary institutions^28^. Obstetric medical institutions are increasingly becoming more centralized due to a shortage of obstetricians in Japan and a decrease in the number of medical institutions providing perinatal care^29^. Therefore, division of roles is increasingly practiced in tertiary institutions and institutions that manage low-risk pregnancies; the latter need to determine when to transfer pregnant women at a high risk of delivering SGA infants to tertiary institutions. Here, our prediction model may provide an early opportunity for the recognition of such women, allowing sufficient time for decision-making on their transfer. However, the NB for each of the risk scores was low in this study. Therefore, it is necessary to create a prediction model for SGA infants that increases NB more than the current model. Additionally, our model (in particular, model 2 during mid-gestation) for predicting preterm SGA infants should be updated though recalibration in the future. We would like to propose the use of a risk score for predicting SGA infants, as a supportive rather than a mandatory tool, at prenatal checkups. The decision to use our risk score for predicting SGA infants should be taken by the medical institution concerned.

The strength of this study is that many variables that were used for creating a prediction model for SGA infants were collected prospectively in a large sample size cohort study. Conversely, the limitations of the study are as follows. First, external validation of the prediction model was not performed. Other prediction models for SGA infants, which were constructed in other countries, have been evaluated for external validation^30^. Although several characteristics in this study (including the maternal age; proportions of primipara, preterm births, and low-birth-weight infants; mean gestational age at delivery; and infant birth weight) were similar to those used in the Japan Environment and Children's Study (a nationwide birth cohort study in Japan), we will perform an external validation in the near future^31^. Second, it is unknown whether low maternal birth weight was attributable to SGA or preterm births, because information on the gestational week when the subjects were born in was not collected. Third, data on other predictors of SGA infants, including prenatal ultrasonographic findings on abnormal cord insertion site, and abnormal cord coiling which leads to impaired cord blood flow were not collected in this study^32,33^. Furthermore, neither the maternal uterine artery pulsatility index nor biochemical markers such as the pregnancy-associated plasma protein-A were measured in this study^34,35^. Moreover, because the placental growth factor data were available for only a small number of subjects in this study, this parameter could not be incorporated into the prediction model. Therefore, we could not evaluate predictive performance of other prediction models of SGA infants for subjects in this study. In addition, we also could not compare predictive performance among risk scores in this study and other prediction models. However, these parameters are not routinely measured in clinical practice in Japan. Therefore, our prediction model may be useful in environments where provisions for such skill-intensive techniques and special measurement systems are not available. Riskin-Mashiah, S. et al. reported that fasting plasma glucose (FPG) in the first trimester was a predictor of infant birth weight^36^. However, 86.0% of data on FPG in the first trimester was missing in this study. In addition, the percentage of missing data on family history of DM, a risk factor of gestational diabetes mellitus, was 37.3%. Therefore, neither FPG in the first trimester nor family history of DM could be incorporated into the prediction model due to the high proportion of missing data in this study^37^. Although other parameters of fetal ultrasonography, including biparietal diameter (BPD), abdominal circumference (AC), femur length (FL), and fetal congenital anomalies may improve the predictive ability of our prediction model for SGA infants, there is a high proportion of missing data for at least one of BPD, AC, and FL in this study. Furthermore, neither prenatal ultrasonographic findings on fetal congenital anomalies nor doppler assessment of umbilical artery were recorded in this study. Therefore, we could not include BPD, AC, FL, fetal congenital anomalies, and doppler assessment of umbilical artery in the prediction model. For a sensitivity analysis, the discrimination performance of SGA infants without major congenital anomalies using the prediction model was evaluated. The discrimination performance of SGA infants without major congenital anomalies was similar to that of all SGA infants. The C-statistics and tenfold cross-validated C-statistics of the risk score during early gestation were 0.659 (95% CI: 0.643–0.676) and 0.659 (95% CI: 0.642–0.677), respectively. In model 1 during mid-gestation, the C-statistics and tenfold cross-validated C-statistics were 0.677 (95% CI: 0.661–0.694) and 0.673 (95% CI: 0.661–0.694), respectively. In model 2 during mid-gestation, the C-statistics and tenfold cross-validated C-statistics were 0.723 (95% CI: 0.708–0.738) and 0.723 (95% CI: 0.707–0.739), respectively.

The cause of hypertensive disorders of pregnancy (HDP), especially preeclampsia, is thought to be impaired uterine spiral artery remodeling followed by angiogenic imbalance. As a result, the insufficient uteroplacental perfusion leads to SGA (as a surrogate of FGR)^38^. As low-dose aspirin treatment for pregnant women at high risk of preeclampsia would decrease the risk of delivery of SGA infants, construction of a prediction model for SGA infants with HDP (i.e., preeclampsia) in early gestation will be needed in Japan in the future^39^.

In conclusion, our prediction model for SGA infants, particularly during mid-gestation, may aid in the detection of pregnant women at a high risk of delivering SGA infants in Japan. Further studies for its external validation and improvement of its predictive ability are necessary.

## Methods

### Study design and participants

This study was part of the TMM BirThree Cohort Study, an ongoing prospective cohort study. The TMM BirThree Cohort Study, one of the several cohort studies conducted by the TMM, aimed to 1) monitor the damage to health status due to the Great East Japan Earthquake, 2) study the early diagnosis and treatment of diseases, and 3) perform molecular-epidemiological studies to examine the associations between genetic and environmental factors and diseases^40^.

The TMM BirThree Cohort Study recruited pregnant women (and their family members), whose expected date of confinement was later than February 1, 2014, from obstetric clinics and hospitals in the Miyagi and Iwate Prefectures in Japan from July 19, 2013 to March 31, 2017. Timing of consent to participate in the TMM BirThree Cohort Study was from the whole period of pregnancy to one month after delivery. Written informed consent was obtained from all participants. The study protocol was approved by the Institutional Review Board of the Tohoku University Graduate School of Medicine (approval number: 2013–1-103–1). The study has been conducted in accordance with the Declaration of Helsinki, the Ethical Guidelines for Human Genome/Gene Analysis Research, and all other applicable guidelines^41,42^. The details and cohort profile of the TMM BirThree Cohort Study have been described previously^40,43,44^.

For easy use in clinical settings, we developed a prediction model based on risk scores to predict the delivery of SGA infants, and an internal validation was performed using the data of all the enrolled subjects. This study was also described based on the Transparent Reporting of a multivariable prediction model for Individual Prognosis Or Diagnosis (TRIPOD) statement^45,46^. The official TRIPOD checklist is shown in the supplementary information^46^.

### Candidate explanatory variables of the risk score for predicting SGA infants

The maternal age, height, BMI, weight gain, general medical history and medical history during previous pregnancy, conception method, smoking status, alcohol drinking, clinic blood pressure (BP), standard deviation (SD) of the EFW during mid-gestation, and maternal birth weight were considered as the candidate explanatory variables of the risk score^10,15–17,19,21,34,47–51^. Details on the data collection of the candidate explanatory variables are presented in the supplementary information.

There is a potential for extramaternal survival at ≥ 22 weeks of gestation; furthermore, reference ranges of birth weights are available for Japanese infants from ≥ 22 weeks of gestation onward^52^. Therefore, we considered that a prediction model for SGA infants at < 22 weeks of gestation would be clinically significant for fetal surveillance at the prenatal checkup. Furthermore, the pregnancy period was divided into two periods separated by 18 weeks of gestation, because Japanese reference ranges for the EFW are unavailable for < 18 weeks of gestation^53^. Therefore, we constructed the risk scores to predict SGA infants in the following two gestational periods that were less than 22 weeks: 1) early gestation: 11 weeks, 0 days to 17 weeks, 6 days and 2) mid-gestation: 18 weeks, 0 days to 21 weeks, 6 days.

### Definition of SGA infants

There is a difference in mean infant birth weight between different races^22^. Since 1980, the mean infant birth weight has decreased and the proportion of LBW infants has increased rapidly in Japan^54^. Additionally, owing to the prevalent use of customized infant birth weight percentiles among health practitioners in clinical practice in Japan, we used it to define SGA infants in this study. Data on the infant birth weight, parity, delivery week, and sex were obtained from the medical records, because the infant birth weight percentile in Japan is customized based on these parameters^52,55^. SGA infants were defined as infants whose birth weight was in the < 10^th^ percentile.

### Statistical analyses

Continuous variables of the maternal and neonatal characteristics in this study were expressed as means ± SDs or median (interquartile range), as appropriate. Categorical variables were also expressed as numbers (percentages).

First, we explored the candidate explanatory variables that were associated with SGA infants using a univariate logistic regression model. When the risk score during early gestation was constructed, the maternal age, maternal height, maternal pre-pregnancy BMI, BMI during early gestation, maternal weight gain between pre-pregnancy BW and the initial BW during early gestation, parity, conception method, maternal birth weight, BP during early gestation, smoking status, alcohol consumption, and medical histories of diabetes mellitus (DM), systemic lupus erythematosus (SLE) and/or antiphospholipid syndrome (APS), chronic kidney diseases (CKD), hyperthyroidism, and hypothyroidism were included in the univariate logistic regression model. When the risk score during mid-gestation was constructed, the maternal BMI during mid-gestation, maternal weight gain between initial BW during early gestation and initial BW during mid-gestation, BP during mid-gestation, and SD value of the EFW during mid-gestation were included in a univariate logistic regression model in addition to the explanatory variables that were considered during early gestation. Furthermore, risk scores without EFW during mid-gestation (model 1) and with EFW during mid-gestation (model 2) were created. Explanatory variables that showed a two-sided *P*-value of < 0.20 in the univariate logistic regression model were included in a multiple logistic regression model. Furthermore, the maternal age during early or mid-gestation and height, which were parameters related to SGA infants in previous studies, were also included in the multiple logistic regression model, even when the two-sided *P*-value was ≥ 0.20 in the univariate logistic regression model^15,16^. If the variance inflation factor was greater than 2.5, multicollinearity among several explanatory variables was suspected, and the explanatory variables were either combined or one variable was chosen to decrease the multicollinearity^56^. In the multiple logistic regression model, explanatory variables that contributed to the prediction of SGA infants were selected when each two-sided *P*-value was < 0.05. The sample size in this study satisfied the condition that the number of SGA infants per explanatory variable was 10 or more to avoid overfitting in the multiple logistic regression model^57^.

The regression coefficients, rather than the odds ratio, were divided by the smallest absolute value of the regression coefficients among the selected explanatory variables and then rounded to an integer score^58^. Next, the risk score for predicting SGA infants was calculated by summating the integer score of each explanatory variable.

To assess the discrimination performance of each risk score for the SGA infants, we created receiver operating characteristics (ROC) curves and calculated the C-statistics (also known as the area under the ROC curves). The C-statistics were interpreted as follows: 0.5 (no discrimination); > 0.5 and < 0.7 (poor discrimination); ≥ 0.7 and < 0.8 (acceptable discrimination); ≥ 0.8 and < 0.9 (excellent discrimination); and ≥ 0.9 (outstanding discrimination)^59^. For the internal validation of each risk score, ten-fold cross validation was conducted. Furthermore, a calibration plot using a restricted cubic spline function with four knots was created to assess the calibration^60^. Calibration evaluates the concordance between the predicted probability and observed proportion of the SGA infants. To evaluate the clinical utility of the risk scores for SGA infant prediction by a net benefit (NB), we conducted a decision curve analysis (DCA)^61–63^.

To evaluate the differences in the predictive abilities of the different risk scores for SGA infants, we evaluated the differences in the C-statistics and reclassification. Differences in the C-statistics were determined using the Delong’s test^64^. Reclassification was evaluated by calculating the net reclassification improvement (NRI) and integrated discrimination improvement (IDI)^65–67^. Since there is no established meaningful risk category of SGA infants, we evaluated the continuous NRI (cNRI), rather than the category-based NRI. The overall cNRI evaluates the upward and downward changes in the predicted risk of SGA infants by changing the reference model to the new model. The overall cNRI was calculated as the sum of the event cNRI and nonevent cNRI. The event cNRI indicated the net proportion of subjects who delivered SGA infants and were correctly predicted to have a higher risk of delivering SGA infants. The nonevent cNRI indicated the net proportion of subjects who did not deliver SGA infants and were correctly predicted to have a lower risk of delivering SGA infants. The IDI was calculated by the following formula: IDI = change in the average predicted probability of SGA infants for subjects who delivered SGA infants between the two models—change in the average predicted probability of SGA infants for subjects who had not delivered SGA infants between the two models. When differences in the C-statistics, cNRI, and IDI among the risk scores during early gestation, model 1, and model 2 during mid-gestation were compared, a two-sided *P*-value of < 0.0167 (0.05/3) by the Bonferroni’s correction was considered as statistically significant. In addition, we also compared the decision curves of the risk scores.

Considering that it is unknown whether infants are SGA or not until they are born, we also explored cut-off values with the predicted probability of SGA infants. Thus far, there is no established threshold probability to categorize pregnant women as having a high risk of delivering SGA infants, although the other study reported several cut-off values according to the predicted probability of SGA infants^30^. Therefore, several cut-off values of the risk scores were considered to define a high risk of SGA infant delivery in this study. After the subjects were divided into quintiles based on the distribution of each risk score, the minimum risk scores of the fifth quintile in each risk score were set as the cut-off values. Furthermore, the risk scores closest to threshold probabilities of 0.05, 0.10, 0.15, and 0.20 (i.e., 5%, 10%, 15%, and 20%) were set as the cut-off values for each risk score. The numbers that must be tested corresponding to the threshold probabilities of 0.05, 0.10, 0.15, and 0.20 were 20, 10, 7.7, and 5, respectively. The risk scores which had the maximum Youden index (i.e., sensitivity + specificity—1) were also set as the cut-off values^68^. Then, the true positive rate (i.e., sensitivity), specificity, positive predictive value, negative predictive value, positive likelihood ratio (LR), and negative LR were calculated in each cut-off of the risk score^69^. Furthermore, we calculated each NB when each cut-off risk score was set.

In the sensitivity analysis, we constructed a risk score after multiple imputations by a chained equation (MICE), as described in the Supplementary Material^70^. As an additional analysis, we evaluated the model performance, calibration, and clinical utility of each risk score for predicting preterm and term SGA infants. We also compared the model performance between different risk scores for predicting preterm and term SGA infants.

Statistical software used in the statistical analysis are described in the supplementary information.

## Supplementary Information

Supplementary Information 1.

Supplementary Information 2.

Supplementary Information 3.

Supplementary Information 4.

Supplementary Information 5.

Supplementary Information 6.

Supplementary Information 7.

## Data Availability

The datasets analyzed in this study are available from the corresponding author on reasonable request.
