# Supplementary material for: Risk scores for predicting small for gestational age infants in Japan: The TMM birthree cohort study
Source: Sci Rep. 2022 May 26;12:8921. doi: 10.1038/s41598-022-12892-0 (PMC9135745; doi:10.1038/s41598-022-12892-0)
Supplement: Supplementary file 1 — Supplementary Information 1. [file 41598_2022_12892_MOESM1_ESM.docx]

**Risk Scores for Predicting Small for Gestational Age Infants in Japan:**

**The TMM BirThree Cohort Study**

Noriyuki Iwama, MD, PhD, Taku Obara, Pharm, PhD, Mami Ishikuro, MW, PhD, Keiko Murakami, MPH, PhD, Fumihiko Ueno, Pharm, PhD, Aoi Noda, BPharm, PhD, Tomomi Onuma, Fumiko Matsuzaki, BPharm, Tetsuro Hoshiai, MD, PhD, Masatoshi Saito, MD, PhD, Hirohito Metoki, MD, PhD, Junichi Sugawara, MD, PhD, Nobuo Yaegashi, MD, PhD, and Shinichi Kuriyama, MD, PhD

**Supplementary information**

**Details** **on data collection and categorization of the candidate explanatory variables for constructing risk scores for the prediction of small for gestational age (SGA) infants**

Data on the maternal age during the early and mid-gestational periods, maternal height, parity, and conception method were collected from the medical records. Since prediction model for SGA infants will be used during early and mid-gestation; therefore, we considered appropriate to use the maternal age at that time during pregnancy rather than the maternal age at enrollment in this study.The maternal age during early- and mid-gestation were classified as follows: < 25 years, 25–29.9 years, 30–34.9 years, and ≥ 35 years. The maternal height was divided into quartiles. Parity was classified as follows: primipara, multipara without hypertensive disorders of pregnancy (HDP) or delivery of low birth weight (LBW) infants in a previous pregnancy, and multipara with HDP and/or delivery of LBW infants in a previous pregnancy. The conception method was classified as follows: natural pregnancy, assisted reproductive technology (ART) including conventional *in vitro* fertilization (IVF) and intracytoplasmic sperm injection (ICSI), non-ART (ovulation induction or artificial insemination by the husband), and others. ART was further subdivided into ART with fresh embryo transfer (ET), ART (conventional IVF or ICSI) with frozen-thawed ET (FET), and ART (conventional IVF or ICSI) without information on the method of ET. Data on the maternal pre-pregnancy body weight (BW) and maternal birth weight were obtained from a questionnaire. The maternal pre-pregnancy body mass index (BMI) was calculated by the following formula: pre-pregnancy BW (kilogram)/(height in meter)^2^; this was then divided into quartiles. The maternal birth weight was classified as follows: “< 2,500 g”, “2,500–2,999 g”, “3,000–3,499 g”, “3,500–3,999 g”, “≥ 4,000 g”, “Unknown”, and “No answer”. Due to a small number of subjects in the “≥ 4,000 g” category, the “3,500–3,999 g” and “≥ 4,000 g” categories were combined into the “≥ 3,500 g” category. The “Unknown” and “No answer” categories were also combined.

Data on the medical histories of diabetes mellitus (DM), systemic lupus erythematosus (SLE), antiphospholipid syndrome (APS), chronic kidney diseases (CKD), hyperthyroidism, and hypothyroidism were obtained from the medical records and a questionnaire.

Although the information on the maternal smoking status and alcohol consumption were obtained from two questionnaires, the first questionnaire alone was used. The maternal smoking status was classified as follows: “Never”, “Quit smoking before conception”, “Quit smoking after conception”, “Continued smoking during pregnancy”, and “No answer”. Maternal alcohol consumption was also classified as follows: “Constitutionally never drinker”, “Almost never drinking or quit drinking”, “Continued drinking”, and “No answer”.

Data on the maternal BW, blood pressure (BP), and estimated fetal weight (EFW; in grams), as measured by fetal ultrasound examination at each prenatal checkup, were obtained from the medical records. The maternal BMI at each prenatal checkup was calculated by the following formula: maternal BW (kilogram) at each prenatal checkup/(height in meter)^2^. The BP at each prenatal checkup was categorized based on the Japanese Society of Hypertension Guidelines for The Management of Hypertension (JSH 2019) as follows: “Normal blood pressure (systolic BP [SBP] is < 120 mmHg, and diastolic BP [DBP] is < 80 mmHg)”, “High normal blood pressure (SBP is 120–129 mmHg and DBP is <80 mmHg)”, “Elevated blood pressure (SBP is 130–139 mmHg and/or DBP is 80–89 mmHg)”, and “Grade 1 or higher hypertension (SBP is ≥ 140 mmHg and/or DBP is ≥ 90 mmHg)”. ^1^ The EFW in grams was calculated by the following formula in Japan: EFW in grams = 1.07 × (biparietal diameter in centimeter)^3^ + (abdominal circumference in centimeter)^2^ × femur length in centimeter.^2^ Based on the measured EFW and the Japanese reference values of the mean and standard deviation (SD) of the EFW according to the gestational age, the SD value of the EFW at each prenatal checkup was calculated.^2^ The SD value of the EFW is used for evaluation of fetal growth at prenatal checkup in Japan. In this study, the single SD value of the EFW during mid-gestation was used. The Japan Society of Obstetrics and Gynecology suggests that the approximate threshold value of SD for the EFW for fetal growth restriction diagnosis is ≤ -1.5 SD.^3^ Therefore, the SD value of EFW was categorized per 1-SD as follows: “≤ -1.5 SD”, “> -1.5 SD and ≤ -0.5 SD”, “> -0.5 SD and < +0.5 SD”, “≥ +0.5 SD and < +1.5 SD”, and “≥ +1.5 SD”. Based on a previous study, SD values below -5.0 SD or above +5.0 SD were considered clinically improbable and were excluded.^4^

Because prenatal checkup at < 23 weeks of gestation is conducted every 4 weeks in Japan, several numbers of data are available on the maternal age during early and mid-gestation, maternal BMI during early and mid-gestation, and BP during early and mid-gestation. In this study, the initial data on maternal age, maternal BMI, and BP during early and mid-gestation were used for the analysis. The maternal weight gain between pre-pregnancy BW and initial BW during early gestation was calculated and then divided into quartiles. In addition, maternal weight gain between the initial BW during early gestation and initial BW during mid-gestation was also calculated and divided into quartiles.

Information on chromosomal abnormalities and skeletal dysplasia of the infants were collected from the medical records.

**Multiple imputation by a chained equation (Sensitivity analysis)**

Maternal pre-pregnancy BMI, BMI during early gestation, BMI during mid-gestation, weight gain, and multipara with HDP and/or delivery of LBW infants had missing values owing to missing data on the maternal height, maternal pre-pregnancy BW, maternal BW during early gestation, maternal BW during mid-gestation, and a history of delivery of LBW infants in the previous pregnancy; therefore, these missing data were subjected to multiple imputation by a chained equation (MICE). Ten data sets were created, and each data set was analyzed using univariate and multiple logistic regression models. After the results of each data set were combined, the risk scores during early and mid-gestation were constructed. Both the C-statistics and the 10-fold cross-validated C-statistics of each risk score were then evaluated.

**Statistical software used in the statistical analysis**

We used the SAS software, version 9.4 (SAS Institute Inc., Cary, North Carolina, USA) for data handling and MICE. Furthermore, we also used R, version 4.0.2, to apply a logistic regression model (rms package), perform 10-fold cross validation (cvAUC package), create the receiver operating characteristics curves (pROC package) and calibration plot (rms package and val.prob.ci.2 function), calculate the continuous net reclassification improvement and integrated discrimination improvement (Hmisc package), perform decision curve analysis (rmda package), and perform sensitivity analysis after MICE (psfmi package).^5-11^

**Results of the univariate logistic regression model**

Supplementary Table S1 presents the results of the univariate logistic regression model. The explanatory variables with *P*-values of less than 0.20 were the maternal height, maternal pre-pregnancy BMI, maternal BMI during early and mid-gestation, multipara without HDP or delivery of LBW infants in a previous pregnancy, multipara with HDP and/or delivery of LBW infants in a previous pregnancy, ART with FET, maternal birth weight, history of SLE and/or APS, smoking status (quit smoking after conception, continued smoking during pregnancy, and no answer), Grade 1 or higher of hypertension during early and mid-gestation, and SD value of the EFW during mid-gestation. To avoid strong multicollinearity, the categories of < 25 and 25–29.9 years during early and mid-gestation were combined into one category (i.e., < 30 years).

**Sensitivity analysis using multiple imputation by a chained equation**

As a result of the sensitivity analysis, the selected explanatory variables were the same as those described in supplementary Tables S2, S3, and S4. The C-statistics and 10-fold cross validated C-statistics of each risk score were also similar to those presented in Figure 2. The C-statistics of the risk score during early gestation, in model 1, and in model 2 during mid-gestation were 0.660 (95% CI: 0.644–0.676), 0.680 (95% CI: 0.664–0.696), and 0.729 (95% CI: 0.711–0.744), respectively. In addition, the 10-fold cross validated C-statistics of the risk score during early gestation, in model 1, and in model 2 during mid-gestation were 0.656 (95% CI: 0.640–0.672), 0.677 (95% CI: 0.661–0.692), and 0.726 (95% CI: 0.712–0.741), respectively.

**Additional analysis**

***Definition of preterm and term SGA infants***

We defined preterm SGA infants as infants whose birth weight was in the < 10^th^ percentile and preterm birth from 22 to < 37 weeks of gestation. We also defined term SGA infants as infants whose birth weight was in the < 10^th^ percentile and birth from 37 to < 42 weeks of gestation.

***Number and proportion of preterm and term SGA infants in each category***

As shown in Table 1, the number and proportion of preterm and term SGA infants were 125 (0.7) and 1,001 (5.9), respectively. Supplementary Table S8 shows the number and proportion of non-SGA, preterm SGA, and term SGA infants in each category.

***Model performance and calibration plot of the risk scores for predicting preterm SGA infants***

Supplementary Figure S1 shows the discrimination performance of each risk score for predicting preterm SGA infants. The risk score during early gestation showed a poor discrimination performance. Both model 1 and 2 during mid-gestation showed acceptable discrimination performances. The C-statistics and 10-fold cross-validated C-statistics of model 1 during mid-gestation were 0.713 (95% confidence interval [CI]: 0.671–0.755) and 0.712 (95% CI: 0.689–0.755), respectively. In addition, the C-statistics and 10-fold cross-validated C-statistics of model 2 during mid-gestation were 0.777 (95% confidence interval [CI]: 0.737–0.816) and 0.777 (95% CI: 0.738–0.816), respectively. Supplementary Table S9 shows the observed proportion of term SGA infants and predicted probability of the preterm SGA infants according to the quintiles of each risk score. Supplementary Figure S2 shows the calibration plot of the risk scores for predicting preterm SGA infants. For the risk score during early gestation and model 1 during mid-gestation, the possibility of miscalibration was low. However, model 2 during mid-gestation tended to underestimate the risk of preterm SGA infants (predicted probability was from 0.007 to 0.013), because a calibration plot using a restricted cubic spline function was above the diagonal line (i.e., the line of perfect calibration). As shown in Supplementary Figure S3, the risk score during early gestation, model 1, and model 2 during mid-gestation had a higher net benefit (NB) than that of either all or no subjects considered to be at a high risk of delivering preterm SGA infants when the threshold probabilities were 0.002–0.019 (risk score = -6 to 9), 0.002–0.023 (risk score = -5 to 8), and 0.002–0.037 (risk score = -5 to 9).

***Comparison of model performance between different risk scores for predicting preterm SGA infants***

As shown in Supplementary Table S10, model 2 showed a better discrimination and reclassification for predicting SGA infants during mid-gestation than the risk score during early gestation or model 1 during mid-gestation, except for event net reclassification improvement between model 1 and 2 during mid-gestation. Supplementary Figure S3 also shows that the NB in model 2 during mid-gestation was higher than that in the risk score during early gestation and model 1 during mid-gestation.

***Discrimination and clinical utility based on different cut-off values of the risk scores for prediction of preterm SGA infants***

Supplementary Table S11 shows the discrimination and NB based on different cut-off values of the risk scores for prediction of preterm SGA infants.

***Model performance and calibration plot of the risk scores for predicting term SGA infants***

Supplementary Figure S4 shows the discrimination performance of each risk score for predicting term SGA infants. The risk score during early gestation and model 1 during mid-gestation showed a poor discrimination performance. Models 2 during mid-gestation showed acceptable discrimination performances. The C-statistics and 10-fold cross-validated C-statistics of model 2 during mid-gestation were 0.715 (95% confidence interval [CI]: 0.699–0.731) and 0.715 (95% CI: 0.698–0.732), respectively. Supplementary Table S12 shows the observed proportion of term SGA infants and predicted probability of term SGA infants according to the quintiles of each risk score. Supplementary Figure S5 shows the calibration plot of the risk scores for predicting term SGA infants. The possibility of miscalibration was low, because the calibration curve of each risk score was close to the diagonal line. Supplementary Figure S6 shows the result of decision curve analysis. The risk score during early gestation, model 1, and model 2 during mid-gestation had a higher net benefit (NB) than that of either all or no subjects considered to be at a high risk of delivering term SGA infants when the threshold probabilities were 0.017–0.207 (risk score = -6 to 12), 0.021–0.219 (risk score = -5 to 11), and 0.016–0.265 (risk score = -7 to 11).

***Comparison of model performance between different risk scores for predicting term SGA infants***

As shown in Supplementary Table S13, model 2 showed a better discrimination and reclassification for predicting SGA infants during mid-gestation than the risk score during early gestation or model 1 during mid-gestation. Supplementary Figure S6 also shows that the NB in model 2 during mid-gestation was higher than that in the risk score during early gestation and model 1 during mid-gestation.

***Discrimination and clinical utility based on different cut-off values of the risk scores for prediction of term SGA infants***

Supplementary Table S14 shows the discrimination and NB based on different cut-off values of the risk scores for prediction of term SGA infants.

**References**

1 Umemura, S. *et al.* The Japanese Society of Hypertension Guidelines for the Management of Hypertension (JSH 2019). *Hypertens Res*, doi:10.1038/s41440-019-0284-9 (2019).

2 Okai, T. Standard values of ultrasonic measurements in Japanese fetuses. *Journal of Medical Ultrasonics* **30**, J415-440 (In Japanese) (2003).

3 Minakami, H. *et al.* Guidelines for obstetrical practice in Japan: Japan Society of Obstetrics and Gynecology (JSOG) and Japan Association of Obstetricians and Gynecologists (JAOG) 2014 edition. *J Obstet Gynaecol Res* **40**, 1469-1499, doi:10.1111/jog.12419 (2014).

4 Stirnemann, J. J. *et al.* Implementing the INTERGROWTH-21(st) fetal growth standards in France: a 'flash study' of the College Francais d'Echographie Foetale (CFEF). *Ultrasound Obstet Gynecol* **49**, 487-492, doi:10.1002/uog.17223 (2017).

5 Frank E Harrell Jr (2020). rms: Regression Modeling Strategies. R package version 6.1-0. <https://CRAN.R-project.org/package=rms>.

6 Erin LeDell, Maya Petersen and Mark van der Laan (2014). cvAUC: Cross-Validated Area Under the ROC Curve Confidence Intervals. R package version 1.1.0. <https://CRAN.R-project.org/package=cvAUC>.

7 Robin, X. *et al.* pROC: an open-source package for R and S+ to analyze and compare ROC curves. *BMC bioinformatics* **12**, 77, doi:10.1186/1471-2105-12-77 (2011).

8 Van Calster, B. *et al.* A calibration hierarchy for risk models was defined: from utopia to empirical data. *J Clin Epidemiol* **74**, 167-176, doi:10.1016/j.jclinepi.2015.12.005 (2016).

9 Frank E Harrell Jr, with contributions from Charles Dupont and many others. (2020). Hmisc: Harrell Miscellaneous. R package version 4.4-2. <https://CRAN.R-project.org/package=Hmisc>.

10 Marshall Brown (2018). rmda: Risk Model Decision Analysis. R package version 1.6. <https://CRAN.R-project.org/package=rmda>.

11 Martijn Heymans (2021). psfmi: Prediction Model Selection and Performance Evaluation in Multiple Imputed Datasets. R package version 0.7.1. <https://CRAN.R-project.org/package=psfmi>.

12 TRIPOD Checklist: Prediction Model Development <https://www.tripod-statement.org/resources/>. [accessed 1 January 2021].

**Supplementary Table S1. Results of the univariate logistic regression model**

^a^Regression coefficient

Abbreviations: AIH, artificial insemination by the husband; APS, antiphospholipid syndrome; ART, assisted reproductive technology; BMI, body mass index; BW, body weight; DBP, diastolic blood pressure; EFW, estimated fetal weight; ET, embryo transfer; FET, frozen-thawed ET; HDP, hypertensive disorders of pregnancy; ICSI, intracytoplasmic sperm injection; IVF, *in vitro* fertilization; LBW, low birth weight; SBP, systolic blood pressure; SD, standard deviation; SLE, systemic lupus erythematosus.

| **Explanatory variables** | **OR (95% CI)** | **b**^a^ | ***P*-value** |
| --- | --- | --- | --- |
| **Maternal age during early gestation** |  |  |  |
| < 25 years | 1.00 (Ref.) | - | - |
| 25–29.9 years |  |  |  |
| 30–34.9 years | 0.99 (0.86–1.15) | -0.01 | 0.9 |
| ≥ 35 years | 1.09 (0.93–1.26) | 0.08 | 0.3 |
| **Maternal age during mid-gestation** |  |  |  |
| < 25 years | 1.00 (Ref.) | - | - |
| 25–29.9 years |  |  |  |
| 30–34.9 years | 0.99 (0.87–1.15) | -0.01 | 0.9 |
| ≥ 35 years | 1.09 (0.94–1.27) | 0.09 | 0.3 |
| **Maternal height** |  |  |  |
| Quartile 1 (< 155 cm) | 1.53 (1.28–1.82) | 0.42 | < 0.0001 |
| Quartile 2 (155–158.9 cm) | 1.16  (0.97–1.39) | 0.15 | 0.1 |
| Quartile 3 (159–161.9 cm) | 1.00 (Ref.) | - | - |
| Quartile 4 (≥ 162 cm) | 0.67 (0.55–0.83) | -0.39 | < 0.0001 |
| **Maternal pre-pregnancy BMI** |  |  |  |
| Quartile 1 (< 19.3 kg/m^2^) | 1.49 (1.27–1.75) | 0.40 | < 0.0001 |
| Quartile 2 (≥ 19.3 and < 20.8 kg/m^2^) | 1.07 (0.90–1.27) | 0.06 | 0.4 |
| Quartile 3 (≥ 20.8 and < 23.0 kg/m^2^) | 1.00 (Ref.) | - | - |
| Quartile 4 (≥ 23.0 kg/m^2^) | 0.75 (0.62–0.90) | -0.31 | 0.002 |
| **Initial maternal BMI during early gestation** |  |  |  |
| Quartile 1 (< 19.5 kg/m^2^) | 1.62 (1.37–1.91) | 0.48 | <0.0001 |
| Quartile 2 (≥ 19.5 and < 21.1 kg/m^2^) | 1.22 (1.03–1.46) | 0.20 | 0.03 |
| Quartile 3 (≥ 21.1 and < 23.2 kg/m^2^) | 1.00 (Ref.) | - | - |
| Quartile 4 (≥ 23.2 kg/m^2^) | 0.84 (0.69–1.01) | -0.18 | 0.06 |
| **Initial maternal BMI during mid-gestation** |  |  |  |
| Quartile 1 (< 20.5 kg/m^2^) | 1.75 (1.48–2.07) | 0.57 | < 0.0001 |
| Quartile 2 (≥ 20.5 and < 22.1 kg/m^2^) | 1.28 (1.08–1.53) | 0.25 | 0.01 |
| Quartile 3 (≥ 22.1 and < 24.2 kg/m^2^) | 1.00 (Ref.) | - | - |
| Quartile 4 (≥ 24.2 kg/m^2^) | 0.77 (0.63–0.94) | -0.26 | 0.01 |
| **Weight gain between pre-pregnancy and early gestation**  **(Initial BW during early gestation−pre-pregnancy BW)** |  |  |  |
| Quartile 1 (< -0.6 kg) | 0.97 (0.82–1.15) | -0.03 | 0.7 |
| Quartile 2 (≥ -0.6 and < 0.6 kg) | 1.00 (Ref.) | - | - |
| Quartile 3 (≥ 0.6 and < 1.7 kg) | 0.96  (0.81–1.14) | -0.04 | 0.7 |
| Quartile 4 (≥ 1.7 kg) | 0.95 (0.80–1.12) | -0.06 | 0.5 |
| **Weight gain between early and mid-gestation**  **(Initial BW during mid-gestation−Initial BW during early gestation)** |  |  |  |
| Quartile 1 (< 1.5 kg) | 1.17 (1.10–1.36) | 0.16 | 0.05 |
| Quartile 2 (≥ 1.5 and < 2.4 kg) | 1.00 (Ref.) | - | - |
| Quartile 3 (≥ 2.4 and < 3.4 kg) | 0.71 (0.60–0.84) | -0.35 | < 0.0001 |
| Quartile 4 (≥ 3.4 kg) | 0.55 (0.46–0.66) | -0.60 | < 0.0001 |
| **Parity** |  |  |  |
| Primipara | 1.00 (Ref.) | - | - |
| Multipara without HDP or delivery of LBW infants  in a previous pregnancy | 0.84 (0.74–0.96) | -0.17 | 0.01 |
| Multipara with HDP and/or delivery of LBW infants  in a previous pregnancy | 2.10 (1.75–2.52) | 0.74 | < 0.0001 |
| **Conception method** |  |  |  |
| Natural pregnancy | 1.00 (Ref.) | - | - |
| Non-ART (ovulation induction or AIH) | 1.05 (0.70–1.56) | 0.05 | 0.8 |
| ART (conventional IVF or ICSI) with fresh ET | 0.93 (0.38–2.31) | -0.07 | 0.9 |
| ART (conventional IVF or ICSI) with FET | 0.53 (0.33–0.86) | -0.63 | 0.01 |
| ART (conventional IVF or ICSI) without information  on the method of ET | 1.04 (0.60–1.79) | 0.04 | 0.9 |
| Others | 1.56 (0.47–5.14) | 0.44 | 0.5 |
| **Maternal birth weight** |  |  |  |
| < 2,500 g | 2.51 (1.95–3.24) | 0.92 | < 0.0001 |
| 2,500–2,999 g | 1.87 (1.57–2.23) | 0.63 | < 0.0001 |
| 3,000–3,499 g | 1.00 (Ref.) | - | - |
| ≥ 3,500 g | 0.47 (0.32–0.68) | -0.76 | < 0.0001 |
| Unknown or No answer | 1.42 (1.21–1.61) | 0.35 | < 0.0001 |
| **Medical history** |  |  |  |
| Diabetes mellitus (vs. No) | 0.96 (0.35–2.65) | -0.04 | 0.9 |
| SLE and/or APS (vs. No) | 2.10 (0.73–6.02) | 0.74 | 0.17 |
| Chronic kidney disease (vs. No) | 0.88 (0.28–2.85) | -0.12 | 0.8 |
| Hyperthyroidism (vs. No) | 0.87 (0.47–1.60) | -0.14 | 0.7 |
| Hypothyroidism (vs. No) | 1.17 (0.72–1.89) | 0.15 | 0.5 |
| **Smoking status** |  |  |  |
| Never | 1.00 (Ref.) | - | - |
| Quit smoking before conception | 0.92 (0.79–1.07) | -0.08 | 0.3 |
| Quit smoking after conception | 0.85 (0.70–1.02) | -0.16 | 0.1 |
| Continue smoking during pregnancy | 1.88 (1.38–2.56) | 0.63 | < 0.0001 |
| No answer | 2.13 (1.05–4.31) | 0.75 | 0.04 |
| **Alcohol drinking** |  |  |  |
| Constitutionally never drinker | 1.07 (0.87–1.38) | 0.06 | 0.6 |
| Almost never drinking or quit drinking | 1.00 (Ref.) | - | - |
| Continue drinking during pregnancy | 1.01 (0.87–1.18) | 0.01 | 0.9 |
| No answer | 0.82 (0.26–2.64) | -0.20 | 0.7 |
| **Initial clinic blood pressure level during early gestation** |  |  |  |
| Normal blood pressure  (SBP is < 120 mmHg and DBP is < 80 mmHg) | 1.00 (Ref.) | - | - |
| High normal blood pressure  (SBP is 120–129 mmHg and DBP is < 80 mmHg) | 0.85 (0.71–1.02) | -0.16 | 0.08 |
| Elevated blood pressure  (SBP is 130–139 mmHg and/or DBP is 80–89 mmHg) | 0.93 (0.74–1.16) | -0.08 | 0.5 |
| Grade 1 or higher hypertension  (SBP is ≥ 140 mmHg and/or DBP is ≥ 90 mmHg) | 1.44 (1.03–2.03) | 0.37 | 0.04 |
| **Initial clinic blood pressure level during mid-gestation** |  |  |  |
| Normal blood pressure  (SBP is < 120 mmHg and DBP is < 80 mmHg) | 1.00 (Ref.) | - | - |
| High normal blood pressure  (SBP is 120–129 mmHg and DBP is < 80 mmHg) | 0.91 (0.76–1.08) | -0.10 | 0.3 |
| Elevated blood pressure  (SBP is 130–139 mmHg and/or DBP is 80–89 mmHg) | 0.99 (0.76–1.30) | -0.01 | 1.0 |
| Grade 1 or higher hypertension  (SBP is ≥ 140 mmHg and/or DBP is ≥ 90 mmHg) | 1.67 (1.10–2.52) | 0.51 | 0.02 |
| **Initial SD value of the EFW during mid-gestation** |  |  |  |
| ≥ +1.5 SD | 0.35 (0.25–0.49) | -1.06 | < 0.0001 |
| ≥ +0.5 SD and < +1.5 SD | 0.56 (0.47–0.66) | -0.59 | < 0.0001 |
| > -0.5 SD and < +0.5 SD | 1.00 (Ref.) | - | - |
| > -1.5 SD and ≤ -0.5 SD | 2.34 (2.01–2.71) | 0.85 | < 0.0001 |
| ≤ -1.5 SD | 4.18 (2.89–6.04) | 1.43 | < 0.0001 |

**Supplementary Table S2. Results of the multiple logistic regression model and risk scores during early gestation (11–17 weeks of gestation)**

^a^Regression coefficient

Abbreviations: ART, assisted reproductive technology; BMI, body mass index; CI, confidence interval; DBP, diastolic blood pressure; ET, embryo transfer; FET, frozen-thawed ET; HDP, hypertensive disorders of pregnancy; ICSI, intracytoplasmic sperm injection; IVF, *in vitro* fertilization; LBW, low birth weight; OR, odds ratio; SBP, systolic blood pressure; SD, standard deviation; SGA, small for gestational age.

| **Selected explanatory variables** | **Early gestation (11–17 weeks of gestation)** | | | |
| --- | --- | --- | --- | --- |
|  | **OR (95% CI)** | **b**^a^ | ***P*-value** | **Integer score** |
| **Maternal age during early gestation** |  |  |  |  |
| < 25 years | 1.00 (Ref.) | - | - | 0 |
| 25–29.9 years |  |  |  |  |
| 30–34.9 years |  |  |  |  |
| ≥ 35 years | 1.19 (1.03–1.36) | 0.17 | 0.02 | 1 |
| **Maternal height** |  |  |  |  |
| Quartile 1 (< 155 cm) | 1.31 (1.14–1.51) | 0.27 | < 0.0001 | 2 |
| Quartile 2 (155–158.9 cm) | 1.00 (Ref.) | - | - | 0 |
| Quartile 3 (159–161.9 cm) |  |  |  |  |
| Quartile 4 (≥ 162 cm) | 0.64 (0.54–0.76) | -0.44 | < 0.0001 | -3 |
| **Initial maternal BMI during early gestation** |  |  |  |  |
| Quartile 1 (< 19.5 kg/m^2^) | 1.62 (1.36–1.91) | 0.48 | < 0.0001 | 3 |
| Quartile 2 (≥ 19.5 and < 21.1 kg/m^2^) | 1.22 (1.02–1.46) | 0.20 | 0.03 | 1 |
| Quartile 3 (≥ 21.1 and < 23.2 kg/m^2^) | 1.00 (Ref.) | - | - | 0 |
| Quartile 4 (≥ 23.2 kg/m^2^) | 0.78 (0.64–0.94) | -0.25 | 0.01 | -2 |
| **Parity** |  |  |  |  |
| Primipara | 1.00 (Ref.) | - | - | 0 |
| Multipara without HDP or delivery of LBW infants in a previous pregnancy | 0.85 (0.74–0.97) | -0.16 | 0.02 | -1 |
| Multipara with HDP and/or delivery of LBW infants in a previous pregnancy | 1.85 (1.54–2.24) | 0.62 | < 0.0001 | 4 |
| **Conception method** |  |  |  |  |
| Natural pregnancy or non-ART or ART with fresh ET,  or ART without information on the method of ET, or Others | 1.00 (Ref.) | - | - | 0 |
| ART (conventional IVF or ICSI) with frozen-thawed ET | 0.54 (0.33–0.87) | -0.62 | 0.01 | -4 |
| **Smoking status** |  |  |  |  |
| Never or Quit smoking before conception or Quit smoking after conception or No answer | 1.00 (Ref.) | - | - | 0 |
| Continue smoking during pregnancy | 1.93 (1.41–2.63) | 0.66 | < 0.0001 | 4 |
| **Initial clinic blood pressure level during early gestation** |  |  |  |  |
| Normal blood pressure  (SBP is < 120 mmHg and DBP is < 80 mmHg) | 1.00 (Ref.) | - | - | 0 |
| High normal blood pressure  (SBP is 120–129 mmHg and DBP is < 80 mmHg) |  |  |  |  |
| Elevated blood pressure  (SBP is 130–139 mmHg and/or DBP is 80–89 mmHg) |  |  |  |  |
| Grade 1 or higher hypertension  (SBP is ≥ 140 mmHg and/or DBP is ≥ 90 mmHg) | 1.52 (1.07–2.16) | 0.42 | 0.02 | 3 |
| **Maternal birth weight** |  |  |  |  |
| < 2,500 g | 2.02 (1.56–2.61) | 0.70 | < 0.0001 | 4 |
| 2,500–2,999 g | 1.67 (1.39–1.99) | 0.51 | < 0.0001 | 3 |
| 3,000–3,499 g | 1.00 (Ref.) | - | - | 0 |
| ≥ 3,500 g | 0.52 (0.36–0.76) | -0.65 | < 0.001 | -4 |
| Unknown or No answer | 1.36 (1.16–1.60) | 0.31 | < 0.001 | 2 |
| **Range of total risk scores during early gestation** | **-** | **-** | **-** | -14 to 21 |

**Supplementary Table S3. Results of the multiple logistic regression model and risk score during mid-gestation (Model 1, 18–21 weeks of gestation)**

^a^Regression coefficient

Abbreviations: ART, assisted reproductive technology; BMI, body mass index; BW, body weight; CI, confidence interval; DBP, diastolic blood pressure; ET, embryo transfer; FET, frozen-thawed ET; HDP, hypertensive disorders of pregnancy; ICSI, intracytoplasmic sperm injection; IVF, *in vitro* fertilization; LBW, low birth weight; OR, odds ratio; SBP, systolic blood pressure; SD, standard deviation; SGA, small for gestational age.

| **Selected explanatory variables** | **Mid-gestation (18–21 weeks of gestation)** | | | |
| --- | --- | --- | --- | --- |
|  | **Model 1** | | | |
|  | **OR (95% CI)** | **b**^a^ | ***P*-value** | **Integer score** |
| **Maternal age during mid-gestation** |  |  |  |  |
| < 25 years | 1.00 (Ref.) | - | - | 0 |
| 25–29.9 years |  |  |  |  |
| 30–34.9 years |  |  |  |  |
| ≥ 35 years | 1.20 (1.05–1.38) | 0.18 | 0.01 | 1 |
| **Maternal height** |  |  |  |  |
| Quartile 1 (< 155 cm) | 1.29 (1.12–1.49) | 0.26 | < 0.001 | 2 |
| Quartile 2 (155–158.9 cm) | 1.00 (Ref.) | - | - | 0 |
| Quartile 3 (159–161.9 cm) |  |  |  |  |
| Quartile 4 (≥ 162 cm) | 0.64 (0.54–0.75) | -0.45 | < 0.0001 | -3 |
| **Initial maternal BMI during mid-gestation** |  |  |  |  |
| Quartile 1 (< 20.5 kg/m^2^) | 1.68 (1.42–1.99) | 0.52 | < 0.0001 | 3 |
| Quartile 2 (≥ 20.5 and < 22.1 kg/m^2^) | 1.28 (1.07–1.52) | 0.25 | 0.01 | 1 |
| Quartile 3 (≥ 22.1 and < 24.2 kg/m^2^) | 1.00 (Ref.) | - | - | 0 |
| Quartile 4 (≥ 24.2 kg/m^2^) | 0.67 (0.54–0.75) | -0.41 | < 0.0001 | -2 |
| **Weight gain between early and mid-gestation (Initial BW during mid-gestation−Initial BW during early gestation)** |  |  |  |  |
| Quartile 1 (< 1.5 kg) | 1.24 (1.06–1.45) | 0.21 | 0.01 | 1 |
| Quartile 2 (≥ 1.5 and < 2.4 kg) | 1.00 (Ref.) | - | - | 0 |
| Quartile 3 (≥ 2.4 and < 3.4 kg) | 0.73 (0.61–0.87) | -0.32 | < 0.001 | -2 |
| Quartile 4 (≥ 3.4 kg) | 0.61 (0.50–0.74) | -0.50 | < 0.0001 | -3 |
| **Parity** |  |  |  |  |
| Primipara | 1.00 (Ref.) | - | - | 0 |
| Multipara without HDP or delivery of LBW infants in a previous pregnancy | 0.84 (0.74–0.96) | -0.17 | 0.01 | -1 |
| Multipara with HDP and/or delivery of LBW infants in a previous pregnancy | 1.76 (1.46–2.13) | 0.57 | < 0.0001 | 3 |
| **Conception method** |  |  |  |  |
| Natural pregnancy or non-ART   or ART with fresh ET  or ART without information on the method of ET, or Others | 1.00 (Ref.) | - | - | 0 |
| ART (conventional IVF or ICSI) with frozen-thawed ET | 0.50 (0.31–0.81) | -0.70 | 0.005 | -4 |
| **Smoking status** |  |  |  |  |
| Never or Quit smoking before conception   or Quit smoking after conception or No answer | 1.00 (Ref.) | - | - | 0 |
| Continue smoking during pregnancy | 1.95 (1.42–2.66) | 0.67 | < 0.0001 | 4 |
| **Initial clinic blood pressure level during mid-gestation** |  |  |  |  |
| Normal blood pressure  (SBP is < 120 mmHg and DBP is < 80 mmHg) | 1.00 (Ref.) | - | - | 0 |
| High normal blood pressure  (SBP is 120–129 mmHg and DBP is <80 mmHg) |  |  |  |  |
| Elevated blood pressure  (SBP is 130–139 mmHg and/or DBP is 80–89 mmHg) |  |  |  |  |
| Grade 1 or higher hypertension  (SBP is ≥ 140 mmHg and/or DBP is ≥ 90 mmHg) | 1.74 (1.13–2.66) | 0.55 | 0.01 | 3 |
| **Maternal birth weight** |  |  |  |  |
| < 2,500 g | 1.95 (1.51–2.54) | 0.67 | < 0.0001 | 4 |
| 2,500–2,999 g | 1.63 (1.36–1.96) | 0.49 | < 0.0001 | 3 |
| 3,000–3,499 g | 1.00 (Ref.) | - | - | 0 |
| ≥ 3,500 g | 0.53 (0.36–0.77) | -0.64 | 0.001 | -4 |
| Unknown or No answer | 1.35 (1.15–1.58) | 0.30 | < 0.001 | 2 |
| **Range of total risk scores**  **during mid-gestation** | **-** | **-** | **-** | -17 to 21 |

**Supplementary Table S4. Results of the multiple logistic regression model and risk score during mid-gestation (Model 2, 18–21 weeks of gestation)**

^a^Regression coefficient

Abbreviations: ART, assisted reproductive technology; BMI, body mass index; BW, body weight; CI, confidence interval; DBP, diastolic blood pressure; EFW, estimated fetal weight; ET, embryo transfer; FET, frozen-thawed ET; HDP, hypertensive disorders of pregnancy; ICSI, intracytoplasmic sperm injection; IVF, *in vitro* fertilization; LBW, low birth weight; OR, odds ratio; SBP, systolic blood pressure; SD, standard deviation; SGA, small for gestational age.

| **Selected explanatory variables** | **Mid-gestation (18–21 weeks of gestation)** | | | |
| --- | --- | --- | --- | --- |
|  | **Model 2 (Model 1 + EFW)** | | | |
|  | **OR (95% CI)** | **b**^a^ | ***P*-value** | **Integer score** |
| **Maternal age during mid-gestation** |  |  |  |  |
| < 25 years | 1.00 (Ref.) | - | - | 0 |
| 25–29.9 years |  |  |  |  |
| 30–34.9 years |  |  |  |  |
| ≥ 35 years | 1.25 (1.08–1.44) | 0.22 | 0.002 | 1 |
| **Maternal height** |  |  |  |  |
| Quartile 1 (< 155 cm) | 1.26 (1.10–1.46) | 0.24 | 0.001 | 1 |
| Quartile 2 (155–158.9 cm) | 1.00 (Ref.) | - | - | 0 |
| Quartile 3 (159–161.9 cm) |  |  |  |  |
| Quartile 4 (≥ 162 cm) | 0.67 (0.56–0.80) | -0.40 | < 0.0001 | -2 |
| **Initial maternal BMI during mid-gestation** |  |  |  |  |
| Quartile 1 (< 20.5 kg/m^2^) | 1.67 (1.40–1.98) | 0.51 | < 0.0001 | 3 |
| Quartile 2 (≥ 20.5 and < 22.1 kg/m^2^) | 1.29 (1.08–1.54) | 0.26 | 0.005 | 1 |
| Quartile 3 (≥ 22.1 and < 24.2 kg/m^2^) | 1.00 (Ref.) | - | - | 0 |
| Quartile 4 (≥ 24.2 kg/m^2^) | 0.65 (0.53–0.80) | -0.43 | < 0.0001 | -2 |
| **Weight gain between early and mid-gestation (Initial BW during mid-gestation−Initial BW during early gestation)** |  |  |  |  |
| Quartile 1 (< 1.5 kg) | 1.20 (1.03–1.41) | 0.19 | 0.02 | 1 |
| Quartile 2 (≥ 1.5 and < 2.4 kg) | 1.00 (Ref.) | - | - | 0 |
| Quartile 3 (≥ 2.4 and < 3.4 kg) | 0.74 (0.62–0.88) | -0.30 | 0.001 | -2 |
| Quartile 4 (≥ 3.4 kg) | 0.63 (0.52–0.76) | -0.47 | < 0.0001 | -3 |
| **Parity** |  |  |  |  |
| Primipara | 1.00 (Ref.) | - | - | 0 |
| Multipara without HDP or delivery of LBW infants in a previous pregnancy | 0.81 (0.70–0.93) | -0.21 | 0.002 | -1 |
| Multipara with HDP and/or delivery of LBW infants in a previous pregnancy | 1.69 (1.39–2.04) | 0.52 | < 0.0001 | 3 |
| **Conception method** |  |  |  |  |
| Natural pregnancy or non-ART   or ART with fresh ET  or ART without information on the method of ET, or Others | 1.00 (Ref.) | - | - | 0 |
| ART (conventional IVF or ICSI) with frozen-thawed ET | 0.51 (0.31–0.84) | -0.67 | 0.01 | -4 |
| **Smoking status** |  |  |  |  |
| Never or Quit smoking before conception   or Quit smoking after conception or No answer | 1.00 (Ref.) | - | - | 0 |
| Continue smoking during pregnancy | 1.99 (1.45–2.74) | 0.69 | < 0.0001 | 4 |
| **Initial clinic blood pressure level during mid-gestation** |  |  |  |  |
| Normal blood pressure  (SBP is < 120 mmHg and DBP is < 80 mmHg) | 1.00 (Ref.) | - | - | 0 |
| High normal blood pressure  (SBP is 120–129 mmHg and DBP is <80 mmHg) |  |  |  |  |
| Elevated blood pressure  (SBP is 130–139 mmHg and/or DBP is 80–89 mmHg) |  |  |  |  |
| Grade 1 or higher hypertension  (SBP is ≥ 140 mmHg and/or DBP is ≥ 90 mmHg) | 1.59 (1.03–2.46) | 0.46 | 0.04 | 2 |
| **Maternal birth weight** |  |  |  |  |
| < 2,500 g | 1.87 (1.43–2.46) | 0.63 | < 0.0001 | 3 |
| 2,500–2,999 g | 1.60 (1.33–1.92) | 0.47 | < 0.0001 | 3 |
| 3,000–3,499 g | 1.00 (Ref.) | - | - | 0 |
| ≥ 3,500 g | 0.55 (0.38–0.81) | -0.60 | 0.002 | -3 |
| Unknown or No answer | 1.30 (1.11–1.54) | 0.27 | 0.001 | 1 |
| **Initial SD value of the EFW during mid-gestation** |  |  |  |  |
| ≥ +1.5 SD | 0.36 (0.25–0.51) | -1.02 | < 0.0001 | -5 |
| ≥ +0.5 SD and < +1.5 SD | 0.57 (0.48–0.67) | -0.57 | < 0.0001 | -3 |
| > -0.5 SD and < +0.5 SD | 1.00 (Ref.) | - | - | 0 |
| > -1.5 SD and ≤ -0.5 SD | 2.21 (1.90–2.58) | 0.80 | < 0.0001 | 4 |
| ≤ -1.5 SD | 3.72 (2.54–5.44) | 1.31 | < 0.0001 | 7 |
| **Range of total risk scores**  **during mid-gestation** | **-** | **-** | **-** | -20 to 25 |

**Supplementary Table S5. Comparison of the models’ performances between different risk scores for predicting SGA infants**^a^

^a^A two-sided *P*-value of less than 0.0167 (0.05/3) by the Bonferroni correction was considered statistically significant.

Abbreviations: CI, confidence interval; NRI, net reclassification improvement; IDI, integrated discrimination improvement; SGA, small for gestational age.

| **Differences in model performance** | **Mid-gestation (Model 1) vs. Early gestation** | **Mid-gestation (Model 2) vs. Early gestation** | **Mid-gestation (Model 2) vs. Mid-gestation (Model 1)** |
| --- | --- | --- | --- |
| **Difference in the C-statistics (95% CI)** | 0.019 (0.010–0.028) *P*–value < 0.001 | 0.067 (0.053–0.081) *P*–value < 0.0001 | 0.048 (0.037–0.059) *P*–value < 0.0001 |
| **Reclassification** |  |  |  |
| **Continuous NRI** |  |  |  |
| Overall NRI (95% CI) | 0.249 (0.190–0.308) *P*–value < 0.0001 | 0.454 (0.395–0.513) *P*–value < 0.0001 | 0.372 (0.313–0.432) *P*–value < 0.0001 |
| Event NRI (95% CI) | 0.222 (0.165–0.279) *P*–value < 0.0001 | 0.169 (0.111–0.226) *P*–value < 0.0001 | 0.165 (0.108–0.223) *P*–value < 0.0001 |
| Nonevent NRI (95% CI) | 0.027 (0.011–0.042) *P*–value < 0.0001 | 0.285 (0.270–0.300) *P*–value < 0.0001 | 0.207 (0.192–0.222) *P*–value < 0.0001 |
| **IDI (95% CI)** | 0.007 (0.005–0.009) *P*–value < 0.0001 | 0.031 (0.027–0.036) *P*–value < 0.0001 | 0.024 (0.021–0.028) *P*–value < 0.0001 |

**Supplementary Table S6. Discrimination performance and the NB when risk scores closest to the threshold probabilities of 0.05, 0.10, 0.15, and 0.20 (i.e., 5%, 10%, 15%, and 20%, respectively) were set as the cut-off values**

Abbreviations: CI, confidence interval; LR, likelihood ratio; NB, net benefit; NPV, negative predictive value; PPV, positive predictive value; SGA, small for gestational age; TPR, true positive rate.

| **Cut-off** | **TPR (Sensitivity) (95% CI)** | **Specificity (95% CI)** | **PPV (95% CI)** | **NPV (95% CI)** | **Positive LR (95% CI)** | **Negative LR (95% CI)** | **NB** |
| --- | --- | --- | --- | --- | --- | --- | --- |
| **Threshold probability of SGA infants closest to 0.05 (5%)** |  |  |  |  |  |  |  |
| **Early gestation** |  |  |  |  |  |  |  |
| Risk score = 1   (Predicted probability  of SGA infants = 0.053) | 0.801 (0.778–0.824) | 0.393 (0.385–0.400) | 0.085 (0.080–0.090) | 0.965 (0.961–0.970) | 1.32 (1.28–1.36) | 0.51 (0.45–0.57) | 0.022 |
| **Mid-gestation** |  |  |  |  |  |  |  |
| Model 1: Risk score = 0  (Predicted probability  of SGA infants = 0.051) | 0.815 (0.793–0.838) | 0.403 (0.396–0.411) | 0.088 (0.083–0.093) | 0.969 (0.964–0.973) | 1.37 (1.33–1.41) | 0.46 (0.41–0.52) | 0.024 |
| Model 2: Risk score = -1  (Predicted probability  of SGA infants = 0.047) | 0.825 (0.803–0.847) | 0.457 (0.450–0.465) | 0.097 (0.091–0.103) | 0.974 (0.970–0.977) | 1.52 (1.48–1.57) | 0.38 (0.34–0.44) | 0.030 |
| **Threshold probability of SGA infants closest to 0.10 (10%)** |  |  |  |  |  |  |  |
| **Early gestation** |  |  |  |  |  |  |  |
| Risk score = 5  (Predicted probability  of SGA infants = 0.094) | 0.401 (0.373–0.430) | 0.800 (0.791–0.803) | 0.122 (0.112–0.133) | 0.950 (0.946–0.953) | 1.98 (1.83–2.14) | 0.75 (0.72–0.79) | 0.007 |
| **Mid-gestation** |  |  |  |  |  |  |  |
| Model 1: Risk score = 4  (Predicted probability  of SGA infants = 0.095) | 0.447 (0.418–0.476) | 0.790 (0.784–0.797) | 0.131 (0.129–0.141) | 0.953 (0.949–0.957) | 2.13 (1.98–2.29) | 0.70 (0.66–0.74) | 0.009 |
| Model 2: Risk score = 3  (Predicted probability   of SGA infants = 0.094) | 0.515 (0.486–0.544) | 0.787 (0.780–0.793) | 0.146 (0.135–0.157) | 0.958 (0.955–0.962) | 2.42 (2.27–2.58) | 0.62 (0.58–0.66) | 0.013 |
| **Threshold probability of SGA infants closest to 0.15 (15%)** |  |  |  |  |  |  |  |
| **Early gestation** |  |  |  |  |  |  |  |
| Risk score = 8  (Predicted probability  of SGA infants = 0.141) | 0.135 (0.115–0.155) | 0.952 (0.949–0.955) | 0.166 (0.142–0.190) | 0.940 (0.936–0.943) | 2.81 (2.39–3.31) | 0.91 (0.89–0.93) | 0.002 |
| **Mid-gestation** |  |  |  |  |  |  |  |
| Model 1: Risk score = 7  (Predicted probability  of SGA infants = 0.148) | 0.184 (0.161–0.206) | 0.944 (0.940–0.947) | 0.188 (0.164–0.211) | 0.942 (0.939–0.946) | 3.27 (2.85–3.75) | 0.87 (0.84–0.89) | 0.003 |
| Model 2: Risk score = 6  (Predicted probability  of SGA infants = 0.152) | 0.275 (0.249–0.301) | 0.923 (0.919–0.927) | 0.201 (0.181–0.221) | 0.949 (0.945–0.952) | 3.56 (3.20–3.97) | 0.76 (0.76–0.81) | 0.005 |
| **Threshold probability of SGA infants closest to 0.20 (20%)** |  |  |  |  |  |  |  |
| **Early gestation** |  |  |  |  |  |  |  |
| Risk score = 11  (Predicted probability  of SGA infants = 0.208) | 0.033 (0.022–0.043) | 0.991 (0.990–0.993) | 0.209 (0.149–0.269) | 0.936 (0.932–0.939) | 3.74 (2.62–5.35) | 0.94 (0.93–0.94) | 0.0004 |
| **Mid-gestation** |  |  |  |  |  |  |  |
| Model 1: Risk score = 9  (Predicted probability  of SGA infants = 0.195) | 0.070 (0.055–0.085) | 0.983 (0.981–0.985) | 0.229 (0.185–0.273) | 0.937 (0.934–0.941) | 4.21 (3.30–5.37) | 0.95 (0.93–0.96) | 0.0004 |
| Model 2: Risk score = 8  (Predicted probability  of SGA infants = 0.207) | 0.168 (0.146–0.190) | 0.966 (0.963–0.969) | 0.260 (0.228–0.292) | 0.943 (0.939–0.946) | 4.98 (4.26–5.81) | 0.86 (0.84–0.88) | 0.002 |

**Supplementary Table S7. Discrimination performance and the NB when risk score which has the maximum Youden index were set as the cut-off value.**

Abbreviations: CI, confidence interval; LR, likelihood ratio; NB, net benefit; NPV, negative predictive value; PPV, positive predictive value; SGA, small for gestational age; TPR, true positive rate.

| **Cut-off (Risk score which has the maximum Youden index)** | **TPR (Sensitivity) (95% CI)** | **Specificity (95% CI)** | **PPV (95% CI)** | **NPV (95% CI)** | **Positive LR (95% CI)** | **Negative LR (95% CI)** | **NB** |
| --- | --- | --- | --- | --- | --- | --- | --- |
| **Early gestation** |  |  |  |  |  |  |  |
| Risk score = 4  (Predicted probability   of SGA infants = 0.082) | 0.513 (0.484–0.543) | 0.717 (0.710–0.724) | 0.114 (0.105–0.122) | 0.954 (0.951–0.958) | 1.82 (1.71–1.93) | 0.68 (0.64–0.72) | 0.010 |
| **Mid–gestation** |  |  |  |  |  |  |  |
| Model 1: Risk score = 2  (Predicted probability   of SGA infants = 0.070) | 0.638 (0.610–0.666) | 0.614 (0.607–0.622) | 0.105 (0.097–0.112) | 0.960 (0.956–0.964) | 1.65 (1.58–1.74) | 0.59 (0.55–0.64) | 0.015 |
| Model 2: Risk score = 1  (Predicted probability   of SGA infants = 0.066) | 0.691 (0.664–0.718) | 0.637 (0.630–0.645) | 0.118 (0.111–0.126) | 0.967 (0.963–0.970) | 1.90 (1.82–1.99) | 0.49 (0.44–0.53) | 0.022 |

**Supplementary Table S8. The number and proportion of non-SGA, preterm SGA, and term SGA infants in each category**

Continuous variables and categorical variables are expressed as mean±SD and number (%), respectively. Abbreviations: AIH, artificial insemination by the husband; APS, antiphospholipid syndrome; ART, assisted reproductive technology; BMI, body mass index; BW, body weight; DBP, diastolic blood pressure; EFW, estimated fetal weight; ET, embryo transfer; FET, frozen-thawed ET; HDP, hypertensive disorders of pregnancy; ICSI, intracytoplasmic sperm injection; IQR, interquartile range; IVF, *in vitro* fertilization; LBW, low birth weight; SBP, systolic blood pressure; SD, standard deviation; SGA, small for gestational age; SLE, systemic lupus erythematosus.

| **Variables** | **The numbers and proportion of non-SGA infants in each category,  cases/number (%)** | **The numbers and proportion of preterm SGA infants in each category,  cases/number (%)** | **The numbers and proportion of term SGA infants in each category,  cases/number (%)** |
| --- | --- | --- | --- |
| **Maternal characteristics** |  |  |  |
| **Age during early gestation, years** |  | **-** | **-** |
| < 25 years, n (%) | 1,404/1,496 (93.9) | 3/1,496 (0.2) | 89/1,496 (6.0) |
| 25–29.9 years, n (%) | 4,377/4,685 (93.4) | 30/4,685 (0.6) | 278/4,685 (5.9) |
| 30–34.9 years, n (%) | 41/6,271 (0.7) | 41/6,271 (0.7) | 362/6,271 (5.8) |
| ≥ 35 years, n (%) | 51/4,621 (1.1) | 51/4,621 (1.1) | 272/4,621 (5.9) |
| **Age during mid-gestation, years** |  | **-** |  |
| < 25 years, n (%) | 1,326/1,412 (93.9) | 3/1,412 (0.2) | 83/1,412 (5.9) |
| 25–29.9 years, n (%) | 4,307/4,609 (93.5) | 28/4,609 (0.6) | 274/4,609 (5.9) |
| 30–34.9 years, n (%) | 5,871/6,275 (93.6) | 41/6,275 (0.7) | 363/6,275 (5.8) |
| ≥ 35 years, n (%) | 4,443/4,777 (93.0) | 53/4,777 (1.1) | 281/4,777 (5.9) |
| **Height, cm** |  | **-** | **-** |
| Quartile 1 (< 155 cm), n (%) | 3,748/4,122 (90.9) | 44/4,122 (1.1) | 330/4,122 (8.0) |
| Quartile 2 (155–158.9 cm), n (%) | 4,634/4,986 (92.9) | 40/4,986 (0.8) | 312/4,986 (6.3) |
| Quartile 3 (159–161.9 cm), n (%) | 3,140/3,345 (93.9) | 23/3,345 (0.7) | 182/3,345 (5.4) |
| Quartile 4 (≥ 162 cm), n (%) | 4,425/4,620 (95.8) | 18/4,620 (0.4) | 177/4,620 (3.8) |
| **Pre-pregnancy BMI, kg/m^2^** |  | **-** | **-** |
| Quartile 1 (< 19.3 kg/m^2^), n (%) | 3,858/4,237 (91.1) | 25/4,237 (0.6) | 354/4,237 (8.4) |
| Quartile 2 (≥ 19.3 and < 20.8 kg/m^2^), n (%) | 3,795/4,063 (93.4) | 30/4,063 (0.7) | 238/4,063 (5.9) |
| Quartile 3 (≥ 20.8 and < 23.0 kg/m^2^), n (%) | 4,186/4,462 (93.8) | 35/4,462 (0.8) | 241/4,462 (5.4) |
| Quartile 4 (≥ 23.0 kg/m^2^), n (%) | 4,108/4,311 (95.3) | 35/4,311 (0.8) | 168/4,311 (3.9) |
| **Initial BMI during early gestation, kg/m^2^** |  | **-** | **-** |
| Quartile 1 (< 19.5 kg/m^2^), n (%) | 3,882/4,263 (91.1) | 27/4,263 (0.6) | 354/4,263 (8.3) |
| Quartile 2 (≥ 19.5 and < 21.1 kg/m^2^), n (%) | 3,925/4,217 (93.1) | 29/4,217 (0.7) | 263/4,217 (6.2) |
| Quartile 3 (≥ 21.1 and < 23.2 kg/m^2^), n (%) | 3,983/4,225 (94.3) | 33/4,225 (0.8) | 209/4,225 (5.0) |
| Quartile 4 (≥ 23.2 kg/m^2^), n (%) | 4,157/4,368 (95.2) | 36/4,368 (0.8) | 175/4,368 (4.0) |
| **Initial BMI during mid-gestation, kg/m^2^** |  | **-** | **-** |
| Quartile 1 (< 20.5 kg/m^2^), n (%) | 3,714/4,101 (90.6) | 30/4,101 (0.7) | 357/4,101 (8.7) |
| Quartile 2 (≥ 20.5 and < 22.1 kg/m^2^), n (%) | 3,960/4,263 (92.9) | 32/4,263 (0.8) | 271/4,263 (6.4) |
| Quartile 3 (≥ 22.1 and < 24.2 kg/m^2^), n (%) | 4,131/4,377 (94.4) | 31/4,377 (0.7) | 215/4,377 (4.9) |
| Quartile 4 (≥ 24.2 kg/m^2^), n (%) | 4,142/4,332 (95.6) | 32/4,332 (0.7) | 158/4,332 (3.7) |
| **Weight gain between pre-pregnancy BW and BW during early gestation  (Initial BW during early gestation - pre-pregnancy BW), kg** |  | **-** | **-** |
| Quartile 1 (< -0.6 kg), n (%) | 4,082/4,370 (93.4) | 26/4,370 (0.6) | 262/4,370 (6.0) |
| Quartile 2 (≥ -0.6 and < 0.6 kg), n (%) | 3,968/4,257 (93.2) | 39/4,257 (0.9) | 250/4,257 (5.9) |
| Quartile 3 (≥ 0.6 and < 1.7 kg), n (%) | 3,818/4,086 (93.4) | 32/4,086 (0.8) | 236/4,086 (5.8) |
| Quartile 4 (≥ 1.7 kg), n (%) | 4,079/4,360 (93.6) | 28/4,360 (0.6) | 253/4,360 (5.8) |
| **Weight gain between BW during early and BW during mid-gestation  (Initial BW during mid-gestation - Initial BW during early gestation), kg** |  | **-** | **-** |
| Quartile 1 (< 1.5 kg), n (%) | 3,737/4,098 (91.2) | 621/4,098 (1.5) | 299/4,098 (7.3) |
| Quartile 2 (≥ 1.5 and < 2.4 kg), n (%) | 4,181/4,527 (92.4) | 29/4,527 (0.6) | 317/4,527 (7.0) |
| Quartile 3 (≥ 2.4 and < 3.4 kg), n (%) | 4,093/4,333 (94.5) | 18/4,333 (0.4) | 222/4,333 (5.1) |
| Quartile 4 (≥ 3.4 kg), n (%) | 3,936/4,115 (95.7) | 16/4,115 (0.4) | 163/4,115 (4.0) |
| **Parity, n (%)** |  |  |  |
| Primipara | 7,544/8,073 (93.5) | 68/8,073 (0.8) | 461/8,073 (5.7) |
| Multipara without a HDP or delivery of LBW infants in the past pregnancy | 7,249/7,676 (94.4) | 33/7,676 (0.4) | 394/7,676 (5.1) |
| Multipara with a HDP and/or delivery of LBW infants in the past pregnancy | 1,154/1,324 (87.2) | 24/1,324 (1.8) | 146/1,324 (11.0) |
| **Conception method, n (%)** |  |  |  |
| Natural pregnancy | 14,824/15,883 (93.3) | 113/15,883 (0.7) | 946/15,883 (6.0) |
| Non-ART | 361/388 (93.0) | 5/388 (1.3) | 22/388 (5.7) |
| ART (conventional IVF or ICSI) with fresh ET | 75/80 (93.8) | 0/80 (0.0) | 5/80 (6.3) |
| ART (conventional IVF or ICSI) with FET | 471/489 (96.3) | 3/489 (0.6) | 15/489 (3.1) |
| ART (conventional IVF or ICSI) without information on the method of ET | 189/203 (93.1) | 3/203 (1.5) | 11/203 (5.4) |
| Others | 27/30 (90.0) | 1/30 (3.3) | 2/30 (6.7) |
| **Maternal birth weight, n (%)** |  |  |  |
| < 2,500 g | 692/783 (88.4) | 9/783 (1.2) | 82/783 (10.5) |
| 2,500–2,999 g | 2,878/3,160 (91.1) | 31/3,160 (1.0) | 251/3,160 (7.9) |
| 3,000–3,499 g | 4,680/4,925 (95.0) | 24/4,925 (0.5) | 221/4,925 (4.5) |
| ≥ 3,500 g | 1,267/1,298 (97.6) | 0/1,298 (0.0) | 31/1,298 (2.4) |
| Unknown or No answer | 6,430/6,907 (93.1) | 61/6,907 (0.9) | 416/6,907 (6.0) |
| **Medical history, n (%)** |  |  |  |
| Diabetes mellitus |  |  |  |
| No | 15,888/17,071 (93.4) | 125/17,071 (0.7) | 997/17,071 (5.9) |
| Yes | 59/63 (93.6) | 0/63 (0.0) | 4/63 (6.4) |
| SLE and/or APS |  |  |  |
| No | 15,920/17,042 (93.4) | 122/17,042 (0.7) | 1,000/17,042 (5.9) |
| Yes | 27/31 (87.1) | 3/31 (9.7) | 1/31 (3.2) |
| Chronic kidney disease |  |  |  |
| No | 15,899/17,022 (93.4) | 123/17,022 (0.7) | 1,000/17,022 (5.9) |
| Yes | 48/51 (94.1) | 2/51 (3.9) | 1/51 (2.0) |
| Hyperthyroidism |  |  |  |
| No | 15,768/16,683 (93.4) | 125/16,683 (0.7) | 990/16,683 (5.9) |
| Yes | 179/190 (94.2) | 0/190 (0.0) | 11/190 (5.8) |
| Hypothyroidism |  |  |  |
| No | 15,728/16,836 (93.4) | 123/16,836 (0.7) | 985/16,836 (5.9) |
| Yes | 219/237 (92.4) | 2/237 (0.8) | 16/237 (6.8) |
| **Smoking status, n (%)** |  |  |  |
| Never | 9,519/10,202 (93.3) | 71/10,202 (0.7) | 612/10,202 (6.0) |
| Quit smoking before conception | 3,755/4,003 (93.8) | 38/4,003 (1.0) | 210/4,003 (5.3) |
| Quit smoking after conception | 2,251/2,388 (94.3) | 14/2,388 (0.6) | 123/2,388 (5.2) |
| Continue smoking during pregnancy | 363/412 (88.1) | 2/412 (0.5) | 47/412 (11.4) |
| No answer | 59/68 (86.8) | 0/68 (0.0) | 9/68 (13.2) |
| **Alcohol drinking, n (%)** |  |  |  |
| Constitutionally never drinker | 921/990 (93.0) | 6/990 (0.6) | 63/990 (6.4) |
| Almost never drinking or Quit drinking | 11,902/12,738 (93.4) | 99/12,738 (0.8) | 737/12,738 (5.8) |
| Continue drinking during pregnancy | 3,072/3,290 (93.4) | 20/3,290 (0.6) | 198/3,290 (6.0) |
| No answer | 52/55 (94.6) | 0/55 (0.0) | 3/55 (5.5) |
| **Initial clinic blood pressure level during early gestation** |  |  |  |
| Normal blood pressure  (SBP is < 120 mmHg and DBP is < 80 mmHg) | 11,872/12,726 (93.3) | 84/12,726 (0.7) | 770/12,726 (6.1) |
| High normal blood pressure  (SBP is 120–129 mmHg and DBP is < 80 mmHg) | 2,375/2,520 (94.3) | 13/2,520 (0.5) | 132/2,520 (5.2) |
| Elevated blood pressure  (SBP is 130–139 mmHg and/or DBP is 80–89 mmHg) | 1,334/1,423 (93.8) | 18/1,423 (1.3) | 71/1,423 (5.0) |
| Grade 1 or higher hypertension  (SBP is ≥ 140 mmHg and/or DBP is ≥ 90 mmHg) | 366/404 (90.6) | 10/404 (2.5) | 28/404 (6.9) |
| **Initial clinic blood pressure level during mid–gestation** |  |  |  |
| Normal blood pressure  (SBP is < 120 mmHg and DBP is < 80 mmHg) | 12,573/13,465 (93.4) | 83/13,465 (0.6) | 809/13,465 (6.0) |
| High normal blood pressure  (SBP is 120–129 mmHg and DBP is < 80 mmHg) | 2,289/2,436 (94.0) | 17/2,436 (0.7) | 130/2,436 (5.3) |
| Elevated blood pressure  (SBP is 130–139 mmHg and/or DBP is 80–89 mmHg) | 865/926 (93.4) | 17/926 (1.8) | 44/926 (4.8) |
| Grade 1 or higher hypertension  (SBP is ≥ 140 mmHg and/or DBP is ≥ 90 mmHg) | 220/246 (89.4) | 8/246 (3.3) | 18/246 (7.3) |
| **Initial SD value of EFW during mid-gestation, SD** | -0.4±0.8 | -0.4±1.0 | -0.1±0.8 |
| ≥ +1.5 SD | 1,406/1,441 (97.6) | 6/1,441 (0.4) | 29/1,441 (2.0) |
| ≥ +0.5 SD and < +1.5 SD | 5,206/5,414 (96.2) | 17/5,414 (0.3) | 191/5,414 (3.5) |
| > -0.5 SD and < +0.5 SD | 7,297/7,821 (93.3) | 43/7,821 (0.6) | 481/7,821 (6.2) |
| > -1.5 SD and ≤ -0.5 SD | 1,908/2,228 (85.6) | 45/2,228 (2.0) | 275/2,228 (12.3) |
| ≤ -1.5 SD | 130/169 (76.9) | 14/169 (8.3) | 25/169 (14.8) |
| **Obstetric complications, n (%)** |  |  |  |
| HDP | 722/846 (85.3) | 54/846 (6.4) | 70/846 (8.3) |
| Gestational diabetes mellitus | 410/438 (93.6) | 2/438 (0.5) | 26/438 (5.9) |
| Placental abruption | 29/34 (85.3) | 3/34 (8.8) | 2/34 (5.9) |
| Placenta previa | 99/102 (97.1) | 1/102 (1.0) | 2/102 (2.0) |
| Low-lying placenta | 66/67 (98.5) | 1/67 (1.5) | 1/67 (1.5) |
| Intrauterine fetal death | 4/8 (50.0) | 1/8 (12.5) | 3/8 (37.5) |
| Stillbirth | 6/12 (50.0) | 5/12 (41.6) | 1/12 (8.3) |
| **Neonatal characteristics** |  |  |  |
| **Infant sex (male/female), n (%)/n (%)** | 8,254/8,837 (93.4) /  7,693/8,236 (93.4) | 66/8,837 (0.8) /  59/8,236 (0.7) | 517/8,837 (5.9) /  484/8,236 (5.9) |
| **Major congenital anomalies** | 293/328 (89.3) | 10/328 (3.1) | 25/328 (7.6) |
| **Chromosomal abnormality** |  |  |  |
| Trisomy 21 | 17/19 (89.5) | 1/19(5.3) | 1/19(5.3) |
| Trisomy 18 | 1/5 (20.0) | 3/5 (60.0) | 1/5 (20.0) |
| Trisomy 13 | 1/2 (50.0) | 0/2 (0.0) | 1/2 (50.0) |
| **Skeletal dysplasia** |  |  |  |
| Thanatophoric dysplasia | 3/3 (100.0) | 0/3 (0.0) | 0/3 (0.0) |
| Achondrogenesis | 1/1 (100.0) | 0/1 (0.0) | 0/1 (0.0) |
| Achondroplasia | 1/1 (100.0) | 0/1 (0.0) | 0/1 (0.0) |
| Osteogenesis imperfecta | - | - | **-** |
| **Other major congenital anomalies** | 273/302 (90.4) | 7/302 (2.3) | 22/302 (7.3) |
| **Gestational age at delivery, weeks** | 39.2±1.6 | 33.0±4.0 | 39.4±1.1 |
| Preterm birth (Delivery at less than 37 weeks of gestation) | 755/880 (85.8) | 125/880 (14.2) | - |
| Preterm birth (Delivery at less than 34 weeks of gestation) | 141/196 (71.9) | 55/196 (28.1) | - |
| Preterm birth (Delivery at less than 32 weeks of gestation) | 956/131 (72.5) | 36/131 (27.5) | - |
| **Infant birth weight** |  |  |  |
| Grams | 3,079±386 | 1,446±537 | 2,462±242 |
| SD value | 0.3±0.9 | -2.0±0.6 | -1.7±0.6 |
| LBW infants (birth weight < 2,500 g), n (%) | 757/1,395 (54.3) | 125/1,395 (9.0) | 513/1,395 (36.8) |

**Supplementary Table S9. Predicted probability and observed proportion of preterm SGA infants according to quintiles of each risk score**

Abbreviations: CI, confidence interval; exp, exponential; SGA, small for gestational age.

| **Total risk score** | **Predicted probability  of preterm SGA infants  (95% CI), %** | **Observed proportion  of preterm SGA infants  (Cases/total), %** |
| --- | --- | --- |
| **Early gestation** |  |  |
| Quintile 1 (≤-2) | 0.3 (0.1–0.5) | 0.3 (9/3,262) |
| Quintile 2 (-1 to 0) | 0.5 (0.2–0.7) | 0.4 (14/3,222) |
| Quintile 3 (1 to 2) | 0.6 (0.3–0.8) | 0.6 (21/3,679) |
| Quintile 4 (3 to 4) | 0.8 (0.5–1.1) | 0.8 (26/3,217) |
| Quintile 5 (≥5, High risk) | 1.4 (1.1–1.9) | 1.5 (55/3,693) |
| Predicted probability of preterm SGA infants based on risk score | exp(logit)/(1 + exp(logit))  where logit = -5.3086 + 0.1512 × (risk score) | - |
| **Model 1 during mid-gestation** |  |  |
| Quintile 1 (≤-3) | 0.2 (0.03–0.3) | 0.2 (6/3,808) |
| Quintile 2 (-2 to -1) | 0.4 (0.1–0.6) | 0.4 (10/2,831) |
| Quintile 3 (0 to 1) | 0.6 (0.3–0.7) | 0.5 (18/3,564) |
| Quintile 4 (2 to 3) | 0.8 (5.9–1.3) | 0.9 (28/3,023) |
| Quintile 5 (≥4, High risk) | 1.6 (1.2–2.0) | 1.6 (63/3,847) |
| Predicted probability of preterm SGA infants based on risk score | exp(logit)/(1 + exp(logit))  where logit = -5.2881 + 0.1915 × (risk score) | - |
| **Model 2 during mid-gestation (Mode 1 + EFW)** |  |  |
| Quintile 1 (≤-5) | 0.1 (0.0–0.2) | 0.1 (3/3,199) |
| Quintile 2 (-4 to -2) | 0.3 (0.1–0.3) | 0.2 (8/4,099) |
| Quintile 3 (-1 to 0) | 0.5 (0.3–0.8) | 0.5 (16/3,017) |
| Quintile 4 (1 to 3) | 0.8 (0.6–1.1) | 0.9 (31/3,538) |
| Quintile 5 (≥4, High risk) | 2.3 (1.7–2.7) | 2.1 (67/3,028) |
| Predicted probability of preterm SGA infants based on risk score | exp(logit)/(1 + exp(logit))  where logit = -5.2917 + 0.2245 × (risk score) | - |

**Supplementary Table S10. Comparison of model performance between different risk scores to predict preterm SGA infants^a^**

^a^A two-sided *P*-value of less than 0.0167 (0.05/3) by the Bonferroni correction was considered statistically significant.

Abbreviations: CI, confidence interval; NRI, net reclassification improvement; IDI, integrated discrimination improvement; SGA, small for gestational age.

| **Difference in model performance** | **Mid-gestation (Model 1) vs. Early gestation** | **Mid-gestation (Model 2) vs. Early gestation** | **Mid-gestation (Model 2) vs. Mid-gestation (Model 1)** |
| --- | --- | --- | --- |
| **Difference in the C-statistics (95% CI)** | 0.052 (0.024–0.081) *P*–value = 0.0004 | 0.116 (0.071–0.161) *P*–value <0.0001 | 0.064 (0.027–0.100) *P*–value = 0.0006 |
| **Reclassification** |  |  |  |
| **Continuous NRI** |  |  |  |
| Overall NRI (95% CI) | 0.538 (0.376–0.700) *P*–value <0.0001 | 0.637 (0.467–0.806) *P*–value <0.0001 | 0.595 (0.422–0.768) *P*–value <0.0001 |
| Event NRI (95% CI) | 0.392 (0.231–0.553) *P*–value <0.0001 | 0.264 (0.095–0.433) *P*–value = 0.0022 | 0.184 (0.012–0.356) *P*–value = 0.0364 |
| Nonevent NRI (95% CI) | 0.146 (0.131–0.161) *P*–value <0.0001 | 0.373 (0.359–0.387) *P*–value <0.0001 | 0.411 (0.397–0.424) *P*–value <0.0001 |
| **IDI (95% CI)** | 0.002 (0.001–0.003) *P*–value <0.0001 | 0.011 (0.007–0.014) *P*–value <0.0001 | 0.024 (0.021–0.028) *P*–value <0.0001 |

**Supplementary Table S11. Discrimination and clinical utility based on different cut-off values of the risk scores for prediction of preterm SGA infants**

^a^Not applicable because there are no preterm SGA infants with a risk score during early gestation of ≥14.

^b^Not applicable because there are no preterm SGA infants with a risk score during early gestation of ≥15.

^c^Not applicable because there are only two preterm SGA infants with a risk score during mid-gestation of ≥12.

Abbreviations: CI, confidence interval; LR, likelihood ratio; NA, not applicable; NB, net benefit; NPV, negative predictive value; PPV, positive predictive value; SGA, small for gestational age; TPR, true positive rate.

| **Cut-off** | **TPR (Sensitivity) (95% CI)** | **Specificity (95% CI)** | **PPV (95% CI)** | **NPV (95% CI)** | **Positive LR (95% CI)** | **Negative LR (95% CI)** | **NB** |
| --- | --- | --- | --- | --- | --- | --- | --- |
| **Minimum risk score of quintile 5** |  |  |  |  |  |  |  |
| **Early gestation** |  |  |  |  |  |  |  |
| Risk score = 5  (Predicted probability   of preterm SGA infants  = 0.010) | 0.440 (0.353–0.527) | 0.785 (0.779–0.792) | 0.015 (0.011–0.019) | 0.785 (0.779–0.792) | 2.05 (1.68–2.50) | 0.71 (0.61–0.83) | 0.001 |
| **Mid-gestation** |  |  |  |  |  |  |  |
| Model 1: Risk score = 4  (Predicted probability   of preterm SGA infants  = 0.011) | 0.504 (0.416–0.592) | 0.777 (0.770–0.783) | 0.016 (0.012–0.020) | 0.995 (0.994–0.996) | 2.26 (1.89–2.69) | 0.64 (0.54–0.76) | 0.001 |
| Model 2: Risk score = 4  (Predicted probability   of preterm SGA infants  = 0.012) | 0.536 (0.449–0.623) | 0.825 (0.820–0.831) | 0.022 (0.017–0.027) | 0.996 (0.995–0.997) | 3.07 (2.60–3.62) | 0.56 (0.47–0.68) | 0.002 |
| **Threshold probability of preterm SGA infants closest to 0.01 (1%)** |  |  |  |  |  |  |  |
| **Early gestation** |  |  |  |  |  |  |  |
| Risk score = 5   (Predicted probability  of preterm SGA infants  = 0.010) | 0.440 (0.353–0.527) | 0.785 (0.779–0.792) | 0.015 (0.011–0.019) | 0.785 (0.779–0.792) | 2.05 (1.68–2.50) | 0.71 (0.61–0.83) | 0.001 |
| **Mid-gestation** |  |  |  |  |  |  |  |
| Model 1: Risk score = 4  (Predicted probability  of preterm SGA infants  = 0.011) | 0.504 (0.416–0.592) | 0.777 (0.770–0.783) | 0.016 (0.012–0.020) | 0.995 (0.994–0.996) | 2.26 (1.89–2.69) | 0.64 (0.54–0.76) | 0.001 |
| Model 2: Risk score = 3  (Predicted probability  of preterm SGA infants  = 0.010) | 0.616 (0.531–0.701) | 0.770 (0.763–0.776) | 0.019 (0.015–0.024) | 0.996 (0.995–0.997) | 2.67 (2.32–3.08) | 0.50 (0.40–0.62) | 0.002 |
| **Threshold probability of preterm SGA infants closest to 0.02 (2%)** |  |  |  |  |  |  |  |
| **Early gestation** |  |  |  |  |  |  |  |
| Risk score = 9  (Predicted probability  of preterm SGA infants  = 0.019) | 0.088 (0.038–0.138) | 0.969 (0.967–0.972) | 0.021 (0.009–0.033) | 0.993 (0.992–0.994) | 2.86 (1.62–5.07) | 0.94 (0.89–0.99) | 0.004 |
| **Mid-gestation** |  |  |  |  |  |  |  |
| Model 1: Risk score = 7  (Predicted probability  of preterm SGA infants  = 0.019) | 0.208 (0.137–0.279) | 0.936 (0.933–0.940) | 0.024 (0.015–0.032) | 0.994 (0.993–0.995) | 3.27 (2.31–4.63) | 0.85 (0.77–0.93) | 0.000 |
| Model 2: Risk score = 6  (Predicted probability   of preterm SGA infants  = 0.019) | 0.376 (0.291–0.461) | 0.912 (0.908–0.916) | 0.030 (0.020–0.039) | 0.995 (0.994–0.996) | 4.26 (3.38–5.37) | 0.68 (0.60–0.78) | 0.001 |
| **Threshold probability of preterm SGA infants closest to 0.03 (3%)** |  |  |  |  |  |  |  |
| **Early gestation** |  |  |  |  |  |  |  |
| Risk score = 12  (Predicted probability  of preterm SGA infants  = 0.030) | 0.016 (0.002–0.057) | 0.995 (0.994–0.996) | 0.024 (0.003–0.085) | 0.993 (0.991–0.994) | 3.39 (0.84–13.6) | 0.99 (0.99–0.99) | 0.000 |
| **Mid-gestation** |  |  |  |  |  |  |  |
| Model 1: Risk score = 9  (Predicted probability  of preterm SGA infants  = 0.028) | 0.064 (0.021–0.107) | 0.980 (0.978–0.982) | 0.023 (0.007–0.039) | 0.993 (0.992–0.994) | 3.22 (1.63–6.35) | 0.96 (0.91–0.99) | 0.0000 |
| Model 2: Risk score = 8  (Predicted probability  of preterm SGA infants  = 0.029) | 0.272 (0.194–0.350) | 0.959 (0.956–0.962) | 0.047 (0.031–0.062) | 0.994 (0.993–0.996) | 6.65 (4.95–8.94) | 0.76 (0.68–0.85) | 0.001 |
| **Threshold probability of preterm SGA infants closest to 0.04 (4%)** |  |  |  |  |  |  |  |
| **Early gestation** |  |  |  |  |  |  |  |
| Risk score = 14  (Predicted probability  of preterm SGA infants  = 0.040) | NA^a^ | NA^a^ | NA^a^ | NA^a^ | NA^a^ | NA^a^ | 0.0000 |
| **Mid-gestation** |  |  |  |  |  |  |  |
| Model 1: Risk score =  11  (Predicted probability  of preterm SGA infants  = 0.040) | 0.016 (0.002–0.057) | 0.995 (0.994–0.996) | 0.025 (0.003–0.087) | 0.993 (0.003–0.087) | 3.48 (0.86–14.0) | 0.99 (0.99–0.99) | 0.0014 |
| Model 2: Risk score = 9  (Predicted probability  of preterm SGA infants  = 0.037) | 0.200 (0.130–0.270) | 0.974 (0.972–0.977) | 0.054 (0.034–0.075) | 0.994 (0.993–0.995) | 7.79 (5.42–11.2) | 0.82 (0.75–0.90) | 0.0010 |
| **Threshold probability of preterm SGA infants closest to 0.05 (5%)** |  |  |  |  |  |  |  |
| **Early gestation** |  |  |  |  |  |  |  |
| Risk score = 15  (Predicted probability  of preterm SGA infants  = 0.046) | NA^b^ | NA^b^ | NA^b^ | NA^b^ | NA^b^ | NA^b^ | 0.0000 |
| **Mid-gestation** |  |  |  |  |  |  |  |
| Model 1: Risk score =  12  (Predicted probability  of preterm SGA infants  = 0.048) | 0.016 (0.002–0.057) | 0.997 (0.996–0.998) | 0.043 (0.005–0.148) | 0.993 (0.992–0.994) | 6.16 (1.51–25.1) | 0.99 (NA^c^) | 0.0000 |
| Model 2: Risk score =  10  (Predicted probability  of preterm SGA infants  = 0.045) | 0.200 (0.130–0.270) | 0.974 (0.972–0.977) | 0.054 (0.034–0.075) | 0.994 (0.993–0.995) | 6.79 (5.42–11.2) | 0.82 (0.75–0.90) | 0.0000 |
| **Risk score which has the maximum Youden index** |  |  |  |  |  |  |  |
| **Early gestation** |  |  |  |  |  |  |  |
| Risk score = 4  (Predicted probability   of preterm SGA infants  = 0.009) | 0.552 (0.465–0.639) | 0.704 (0.697–0.711) | 0.014 (0.010–0.017) | 0.995 (0.994–0.997) | 1.87 (1.59–2.19) | 0.64 (0.52–0.77) | 0.001 |
| **Mid-gestation** |  |  |  |  |  |  |  |
| Model 1: Risk score = 2  (Predicted probability   of preterm SGA infants  = 0.007) | 0.728 (0.650–0.806) | 0.600 (0.593–0.607) | 0.013 (0.011–0.016) | 0.997 (0.996–0.998) | 1.82 (1.63–2.03) | 0.45 (0.34–0.60) | 0.003 |
| Model 2: Risk score = 1  (Predicted probability   of preterm SGA infants  = 0.006) | 0.784 (0.712–0.856) | 0.618 (0.611–0.626) | 0.015 (0.012–0.018) | 0.997 (0.996–0.998) | 2.05 (1.87–2.26) | 0.35 (0.25–0.49) | 0.003 |

**Supplementary Table S12. Predicted probability and observed proportion of term SGA infants according to quintiles of each risk score**

Abbreviations: CI, confidence interval; exp, exponential; SGA, small for gestational age.

| **Total risk score** | **Predicted probability  of term SGA infants  (95% CI), %** | **Observed proportion  of term SGA infants  (Cases/total), %** |
| --- | --- | --- |
| **Early gestation** |  |  |
| Quintile 1 (≤-2) | 2.4 (1.9–2.9) | 2.4 (78/3,262) |
| Quintile 2 (-1 to 0) | 3.9 (3.2–4.5) | 3.8 (123/3,222) |
| Quintile 3 (1 to 2) | 5.1 (4.4–5.8) | 5.1 (187/3,679) |
| Quintile 4 (3 to 4) | 6.7 (5.9–7.6) | 6.7 (216/3,217) |
| Quintile 5 (≥5, High risk) | 10.7 (9.8–11.8) | 10.8 (397/3,693) |
| Predicted probability of term  SGA infants based on risk score | exp(logit)/(1 + exp(logit))  where logit = -3.1577 + 0.1516 × (risk score) | - |
| **Model 1 during mid-gestation** |  |  |
| Quintile 1 (≤-3) | 2.2 (1.8–2.7) | 2.6 (86/3,808) |
| Quintile 2 (-2 to -1) | 3.7 (3.0–4.4) | 3.7 (106/2,831) |
| Quintile 3 (0 to 1) | 5.0 (4.4–5.8) | 5.1 (182/3,564) |
| Quintile 4 (2 to 3) | 6.7 (5.3–7.0) | 6.2 (187/3,023) |
| Quintile 5 (≥4, High risk) | 11.2 (10.4–12.4) | 11.4 (440/3,847) |
| Predicted probability of term  SGA infants based on risk score | exp(logit)/(1 + exp(logit))  where logit = -3.0297 + 0.1599 × (risk score) | - |
| **Model 2 during mid-gestation (Mode 1 + EFW)** |  |  |
| Quintile 1 (≤-5) | 1.7 (1.0–1.8) | 1.4 (48/3,199) |
| Quintile 2 (-4 to -2) | 3.1 (2.8–3.9) | 3.4 (138/4,099) |
| Quintile 3 (-1 to 0) | 4.7 (3.7–5.2) | 4.5 (135/3,017) |
| Quintile 4 (1 to 3) | 6.9 (6.5–8.2) | 7.4 (261/3,538) |
| Quintile 5 (≥4, High risk) | 14.2 (12.6–15.1) | 13.8 (419/3,028) |
| Predicted probability of term  SGA infants based on risk score | exp(logit)/(1 + exp(logit))  where logit = -2.9320 + 0.1738 × (risk score) | - |

**Supplementary Table S13. Comparison of model performance between different risk scores to predict term SGA infants^a^**

^a^A two-sided *P*-value of less than 0.0167 (0.05/3) by the Bonferroni correction was considered statistically significant.

Abbreviations: CI, confidence interval; NRI, net reclassification improvement; IDI, integrated discrimination improvement; SGA, small for gestational age.

| **Difference in model performance** | **Mid-gestation (Model 1) vs. Early gestation** | **Mid-gestation (Model 2) vs. Early gestation** | **Mid-gestation (Model 2) vs. Mid-gestation (Model 1)** |
| --- | --- | --- | --- |
| **Difference in the C-statistics (95% CI)** | 0.014 (0.005–0.024) *P*–value = 0.0004 | 0.059 (0.044–0.074) *P*–value <0.0001 | 0.045 (0.033–0.057) *P–value <0.0001* |
| **Reclassification** |  |  |  |
| **Continuous NRI** |  |  |  |
| Overall NRI (95% CI) | 0.225 (0.162–0.288) *P*–value <0.0001 | 0.418 (0.355–0.481) *P*–value <0.0001 | 0.346 (0.283–0.409) *P*–value <0.0001 |
| Event NRI (95% CI) | 0.203 (0.142–0.264) *P*–value <0.0001 | 0.165 (0.104–0.226) *P*–value <0.0001 | 0.153 (0.092–0.214) *P–value <0.0001* |
| Nonevent NRI (95% CI) | 0.022 (0.007–0.038) *P*–value <0.0001 | 0.373 (0.359–0.387) P–value <0.0001 | 0.193 (0.178–0.208) *P*–value <0.0001 |
| **IDI (95% CI)** | 0.005 (0.004–0.007) *P*–value <0.0001 | 0.023 (0.019–0.027) *P*–value <0.0001 | 0.018 (0.015–0.021) *P*–value <0.0001 |

**Supplementary Table S14. Discrimination and clinical utility based on different cut-off values of the risk scores for prediction of term SGA infants**

Abbreviations: CI, confidence interval; LR, likelihood ratio; NB, net benefit; NPV, negative predictive value; PPV, positive predictive value; SGA, small for gestational age; TPR, true positive rate.

| **Cut-off** | **TPR (Sensitivity) (95% CI)** | **Specificity (95% CI)** | **PPV (95% CI)** | **NPV (95% CI)** | **Positive LR (95% CI)** | **Negative LR (95% CI)** | **NB** |
| --- | --- | --- | --- | --- | --- | --- | --- |
| **Minimum risk score of quintile 5** |  |  |  |  |  |  |  |
| **Early gestation** |  |  |  |  |  |  |  |
| Risk score = 5  (Predicted probability   of term SGA infants =  0.083) | 0.397 (0.366–0.427) | 0.795 (0.789–0.801) | 0.108 (0.098–0.117) | 0.955 (0.951–0.958) | 1.93 (1.78–2.10) | 0.76 (0.72–0.80) | 0.006 |
| **Mid-gestation** |  |  |  |  |  |  |  |
| Model 1: Risk score =  4  (Predicted probability   of term SGA infants =  0.084) | 0.440 (0.409–0.470) | 0.790 (0.784–0.797) | 0.131 (0.120–0.141) | 0.953 (0.949–0.957) | 2.07 (1.92–2.24) | 0.71 (0.67–0.75) | 0.008 |
| Model 2: Risk score =  4  (Predicted probability   of term SGA infants =  0.097) | 0.419 (0.388–0.449) | 0.838 (0.832–0.843) | 0.138 (0.126–0.151) | 0.959 (0.955–0.962) | 2.57 (2.38–2.80) | 0.69 (0.66–0.73) | 0.008 |
| **Threshold probability of term SGA infants closest to 0.05 (5%)** |  |  |  |  |  |  |  |
| **Early gestation** |  |  |  |  |  |  |  |
| Risk score = 1   (Predicted probability  of term SGA infants =  0.047) | 0.799 (0.774–0.824) | 0.391 (0.383–0.398) | 0.076 (0.071–0.081) | 0.969 (0.965–0.973) | 1.31 (1.27–1.36) | 0.51 (0.45–0.58) | 0.019 |
| **Mid-gestation** |  |  |  |  |  |  |  |
| Model 1: Risk score =  1  (Predicted probability  of term SGA infants =  0.054) | 0.722 (0.695–0.750) | 0.512 (0.504–0.519) | 0.088 (0.083–0.093) | 0.967 (0.964–0.971) | 1.48 (1.42–1.54) | 0.54 (0.49–0.60) | 0.016 |
| Model 2: Risk score =  0  (Predicted probability  of term SGA infants =  0.051) | 0.762 (0.736–0.789) | 0.549 (0.541–0.556) | 0.095 (0.089–0.102) | 0.974 (0.970–0.977) | 1.69 (1.63–1.76) | 0.38 (0.34–0.44) | 0.021 |
| **Threshold probability of term SGA infants closest to 0.10 (10%)** |  |  |  |  |  |  |  |
| **Early gestation** |  |  |  |  |  |  |  |
| Risk score = 6  (Predicted probability  of term SGA infants =  0.096) | 0.304 (0.275–0.332) | 0.870 (0.865–0.875) | 0.127 (0.114–0.140) | 0.953 (0.949–0.956) | 2.34 (2.11–2.59) | 0.80 (0.77–0.83) | 0.004 |
| **Mid-gestation** |  |  |  |  |  |  |  |
| Model 1: Risk score =  5  (Predicted probability  of term SGA infants =  0.097) | 0.344 (0.314–0.373) | 0.853 (0.848–0.859) | 0.127 (0.115–0.140) | 0.954 (0.951–0.958) | 2.34 (2.13–2.57) | 0.77 (0.74–0.81) | 0.005 |
| Model 2: Risk score =  4  (Predicted probability   of term SGA infants =  0.097) | 0.419 (0.388–0.449) | 0.838 (0.832–0.843) | 0.138 (0.126–0.151) | 0.959 (0.955–0.962) | 2.57 (2.38–2.80) | 0.69 (0.66–0.73) | 0.008 |
| **Threshold probability of term SGA infants closest to 0.15 (15%)** |  |  |  |  |  |  |  |
| **Early gestation** |  |  |  |  |  |  |  |
| Risk score = 9  (Predicted probability  of term SGA infants =  0.143) | 0.085 (0.068–0.102) | 0.972 (0.970–0.975) | 0.160 (0.129–0.191) | 0.945 (0.941–0.948) | 3.05 (2.44–3.82) | 0.94 (0.92–0.96) | 0.001 |
| **Mid-gestation** |  |  |  |  |  |  |  |
| Model 1: Risk score =  8  (Predicted probability  of term SGA infants =  0.148) | 0.118 (0.098–0.138) | 0.967 (0.964–0.970) | 0.183 (0.153–0.212) | 0.946 (0.943–0.950) | 3.27 (2.85–4.34) | 0.91 (0.89–0.93) | 0.003 |
| Model 2: Risk score =  7  (Predicted probability  of term SGA infants =  0.152) | 0.205 (0.180–0.230) | 0.945 (0.941–0.948) | 0.187 (0.164–0.211) | 0.950 (0.947–0.954) | 3.70 (3.23–4.25) | 0.84 (0.82–0.87) | 0.003 |
| **Threshold probability of term SGA infants closest to 0.20 (20%)** |  |  |  |  |  |  |  |
| **Early gestation** |  |  |  |  |  |  |  |
| Risk score = 12  (Predicted probability  of term SGA infants =  0.208) | 0.020 (0.011–0.029) | 0.996 (0.995–0.997) | 0.244 (0.151–0.337) | 0.942 (0.939–0.946) | 5.18 (3.14–8.54) | 0.98 (0.98–0.99) | 0.0000 |
| **Mid-gestation** |  |  |  |  |  |  |  |
| Model 1: Risk score =  10  (Predicted probability  of term SGA infants =  0.193) | 0.038 (0.026–0.050) | 0.991 (0.989–0.992) | 0.207 (0.148–0.265) | 0.943 (0.939–0.946) | 4.18 (2.94–5.94) | 0.97 (0.96–0.98) | 0.0014 |
| Model 2: Risk score =  9  (Predicted probability  of term SGA infants =  0.203) | 0.104 (0.085–0.123) | 0.978 (0.976–0.980) | 0.226 (0.188–0.264) | 0.946 (0.943–0.949) | 4.69 (3.81–5.78) | 0.92 (0.90–0.94) | 0.0010 |
| **Risk score which has the maximum Youden index** |  |  |  |  |  |  |  |
| **Early gestation** |  |  |  |  |  |  |  |
| Risk score = 4  (Predicted probability   of term SGA infants =  0.072) | 0.508 (0.478–0.539) | 0.715 (0.708–0.722) | 0.100 (0.092–0.108) | 0.959 (0.955–0.963) | 1.79 (1.67–1.91) | 0.69 (0.65–0.73) | 0.009 |
| **Mid-gestation** |  |  |  |  |  |  |  |
| Model 1: Risk score =  3  (Predicted probability   of term SGA infants =  0.072) | 0.534 (0.504–0.565) | 0.704 (0.697–0.711) | 0.101 (0.093–0.109) | 0.960 (0.957–0.964) | 1.81 (1.70–1.92) | 0.66 (0.62–0.71) | 0.010 |
| Model 2: Risk score =  1  (Predicted probability   of term SGA infants =  0.060) | 0.679 (0.650–0.708) | 0.634 (0.626–0.641) | 0.104 (0.096–0.111) | 0.969 (0.966–0.973) | 1.86 (1.77–1.95) | 0.51 (0.46–0.55) | 0.018 |

**The TRIPOD Checklist: Prediction Model Development ^12^**

Abbreviations: NA, not applicable

| **Section/Topic** | **Item** | **Checklist Item** | **Page** |
| --- | --- | --- | --- |
| **Title and abstract** | | | |
| Title | 1 | Identify the study as developing and/or validating a multivariable prediction model, the target population, and the outcome to be predicted. | Title |
| Abstract | 2 | Provide a summary of objectives, study design, setting, participants, sample size, predictors, outcome, statistical analysis, results, and conclusions. | Abstract section |
| **Introduction** | | | |
| Background and objectives | 3a | Explain the medical context (including whether diagnostic or prognostic) and rationale for developing or validating the multivariable prediction model, including references to existing models. | Introduction section |
|  | 3b | Specify the objectives, including whether the study describes the development or validation of the model or both. | Introduction section |
| **Methods** | | | |
| Source of data | 4a | Describe the study design or source of data (e.g., randomized trial, cohort, or registry data), separately for the development and validation data sets, if applicable. | Methods section |
|  | 4b | Specify the key study dates, including start of accrual; end of accrual; and, if applicable, end of follow-up. | Methods section |
| Participants | 5a | Specify key elements of the study setting (e.g., primary care, secondary care, general population) including number and location of centres. | Methods section |
|  | 5b | Describe eligibility criteria for participants. | Figure 1 |
|  | 5c | Give details of treatments received, if relevant. | NA |
| Outcome | 6a | Clearly define the outcome that is predicted by the prediction model, including how and when assessed. | Methods section |
|  | 6b | Report any actions to blind assessment of the outcome to be predicted. | NA |
| Predictors | 7a | Clearly define all predictors used in developing or validating the multivariable prediction model, including how and when they were measured. | Methods section and　supplementary information |
|  | 7b | Report any actions to blind assessment of predictors for the outcome and other predictors. | NA |
| Sample size | 8 | Explain how the study size was arrived at. | Methods section |
| Missing data | 9 | Describe how missing data were handled (e.g., complete-case analysis, single imputation, multiple imputation) with details of any imputation method. | Methods section |
| Statistical analysis methods | 10a | Describe how predictors were handled in the analyses. | Supplementary information |
|  | 10b | Specify type of model, all model-building procedures (including any predictor selection), and method for internal validation. | Methods section |
|  | 10d | Specify all measures used to assess model performance and, if relevant, to compare multiple models. | Methods section and supplementary information |
| Risk groups | 11 | Provide details on how risk groups were created, if done. | Methods section |
| **Results** | | | |
| Participants | 13a | Describe the flow of participants through the study, including the number of participants with and without the outcome and, if applicable, a summary of the follow-up time. A diagram may be helpful. | Results section and Figure 1 |
|  | 13b | Describe the characteristics of the participants (basic demographics, clinical features, available predictors), including the number of participants with missing data for predictors and outcome. | Table1 and supplementary information |
| Model development | 14a | Specify the number of participants and outcome events in each analysis. | Figure 1 and Table 1 |
|  | 14b | If done, report the unadjusted association between each candidate predictor and outcome. | Supplementary information and supplementary Table S1 |
| Model specification | 15a | Present the full prediction model to allow predictions for individuals (i.e., all regression coefficients, and model intercept or baseline survival at a given time point). | Supplementary Tables S3 and S4 |
|  | 15b | Explain how to the use the prediction model. | Table 2 |
| Model performance | 16 | Report performance measures (with CIs) for the prediction model. | Tables 2 and 3, Figures 2–4, supplementary Tables S5–7, and Table S9–14 |
| **Discussion** | | | |
| Limitations | 18 | Discuss any limitations of the study (such as nonrepresentative sample, few events per predictor, missing data). | Discussion section |
| Interpretation | 19b | Give an overall interpretation of the results, considering objectives, limitations, and results from similar studies, and other relevant evidence. | Discussion section |
| Implications | 20 | Discuss the potential clinical use of the model and implications for future research. | Discussion section |
| **Other information** | | | |
| Supplementary information | 21 | Provide information about the availability of supplementary resources, such as study protocol, Web calculator, and data sets. | NA |
| Funding | 22 | Give the source of funding and the role of the funders for the present study. | Acknowledgements section |
