# Supplementary figures and images for: Risk scores for predicting small for gestational age infants in Japan: The TMM birthree cohort study
Source: Sci Rep. 2022 May 26;12:8921. doi: 10.1038/s41598-022-12892-0 (PMC9135745; doi:10.1038/s41598-022-12892-0)

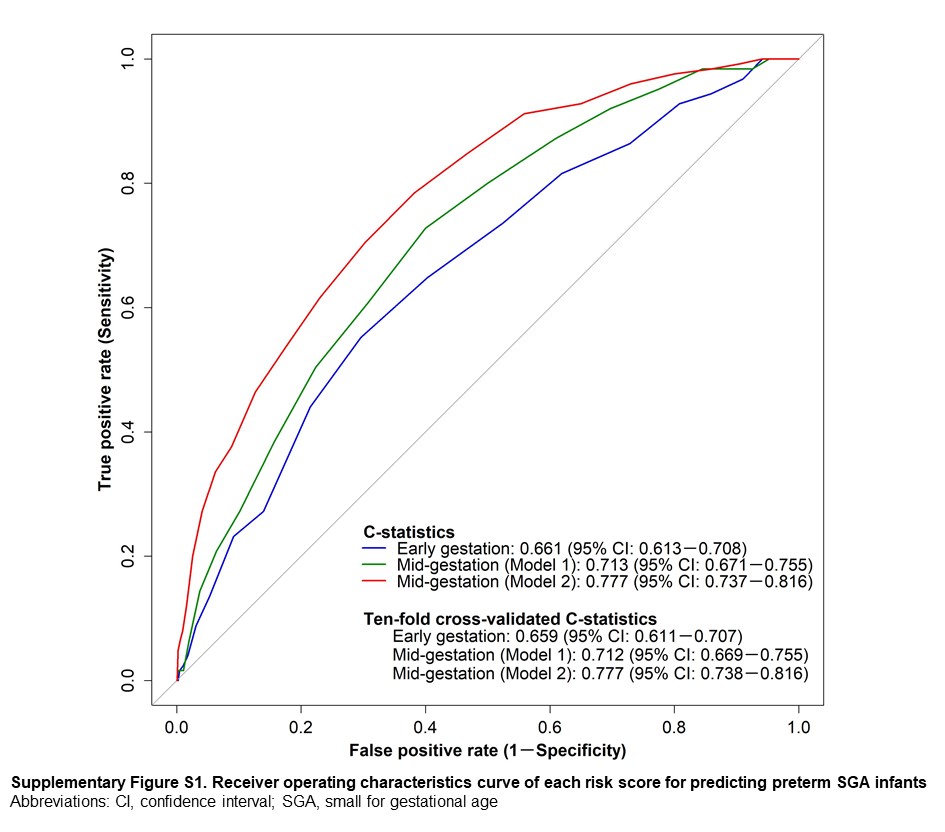

Supplement: Supplementary file 2 — Supplementary Information 2. [file 41598_2022_12892_MOESM2_ESM.jpg]

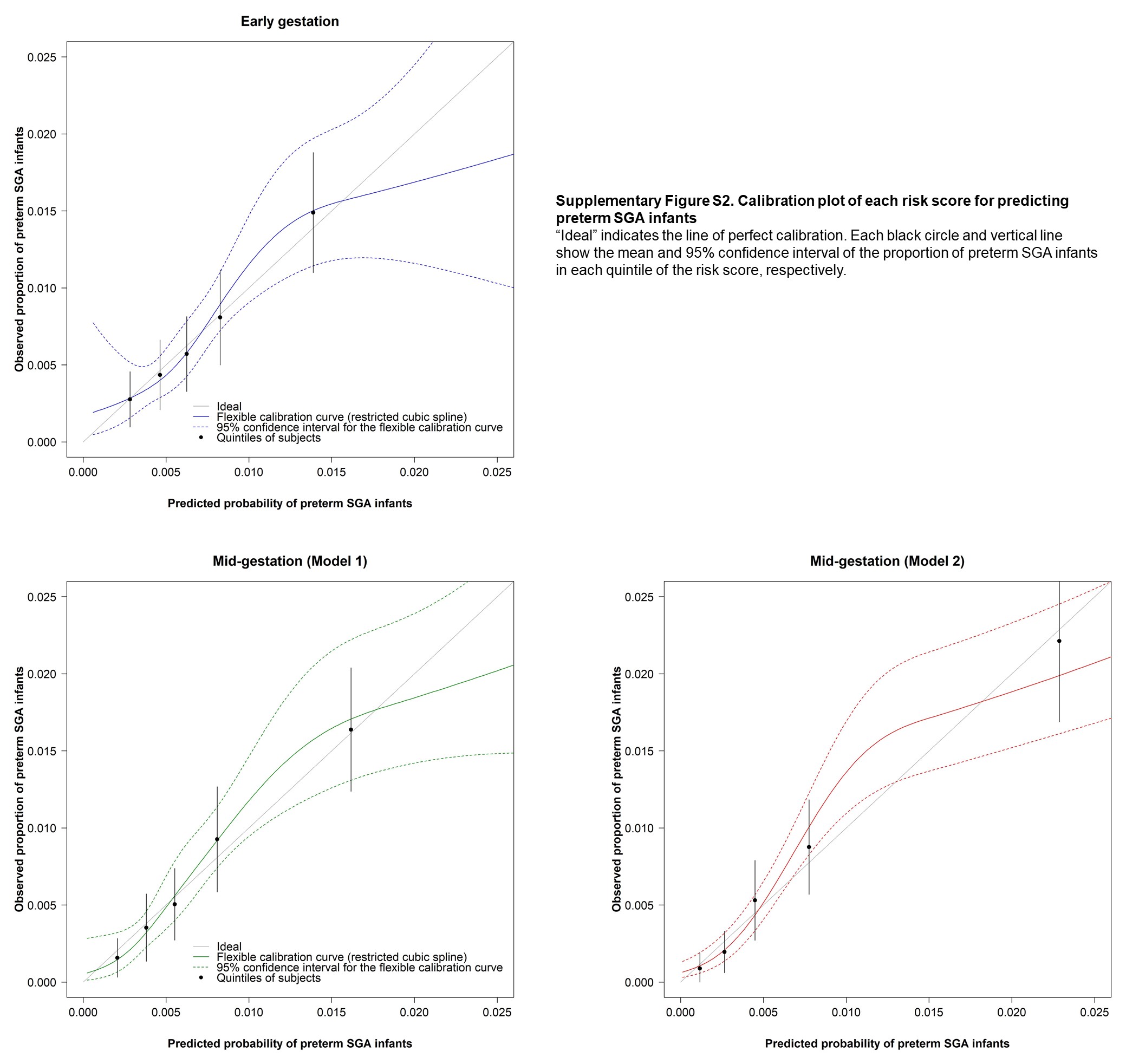

Supplement: Supplementary file 3 — Supplementary Information 3. [file 41598_2022_12892_MOESM3_ESM.jpg]

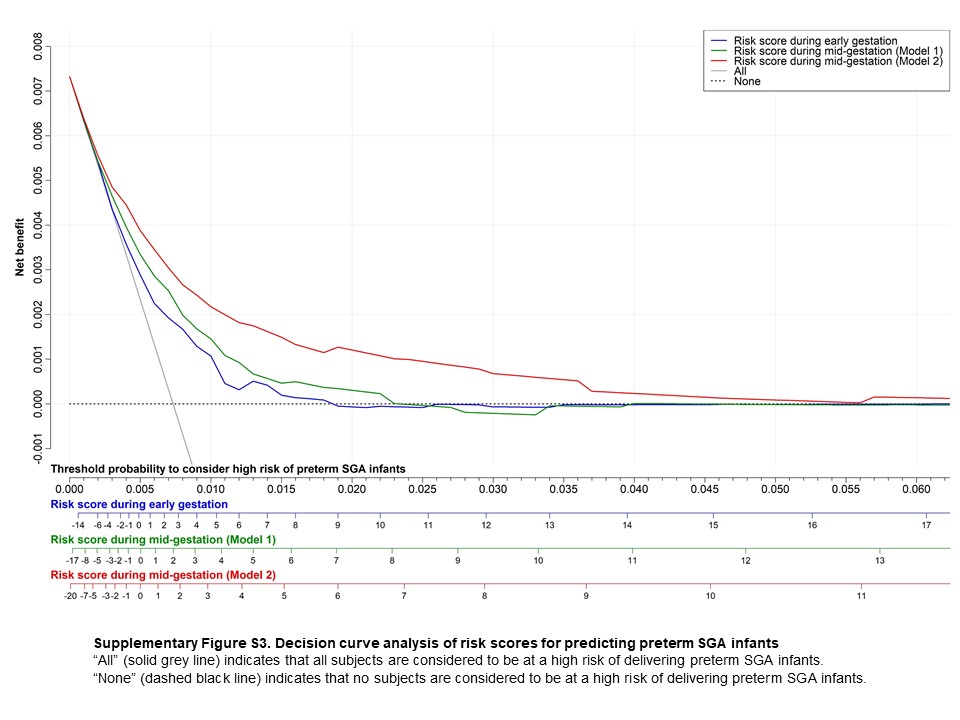

Supplement: Supplementary file 4 — Supplementary Information 4. [file 41598_2022_12892_MOESM4_ESM.jpg]

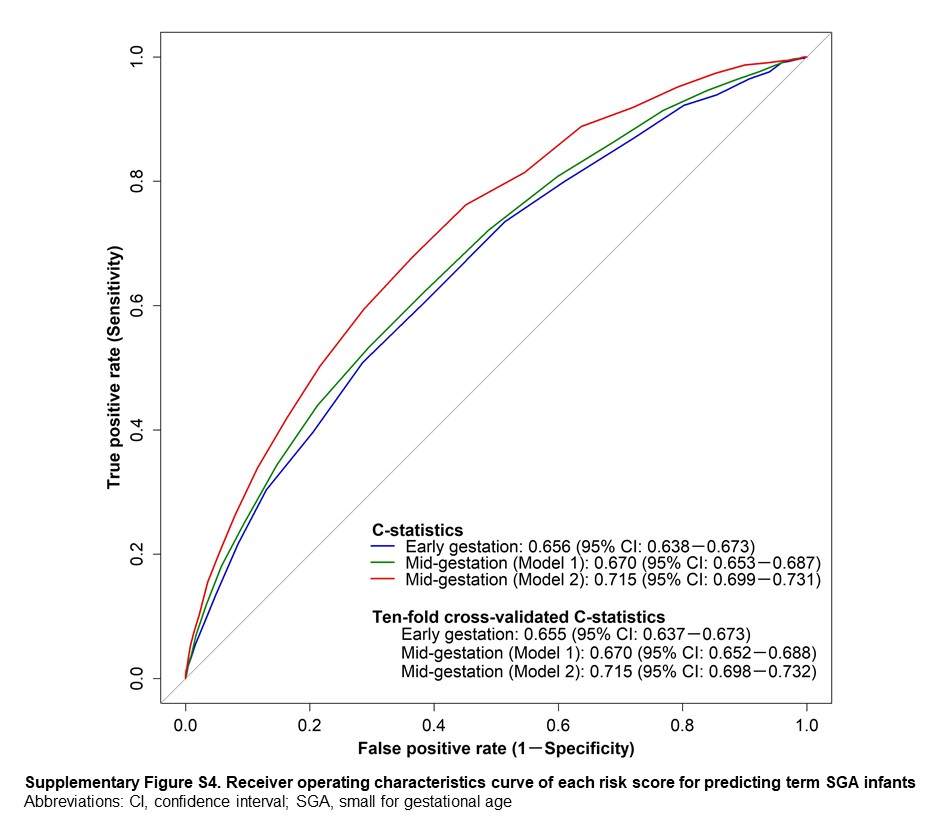

Supplement: Supplementary file 5 — Supplementary Information 5. [file 41598_2022_12892_MOESM5_ESM.jpg]

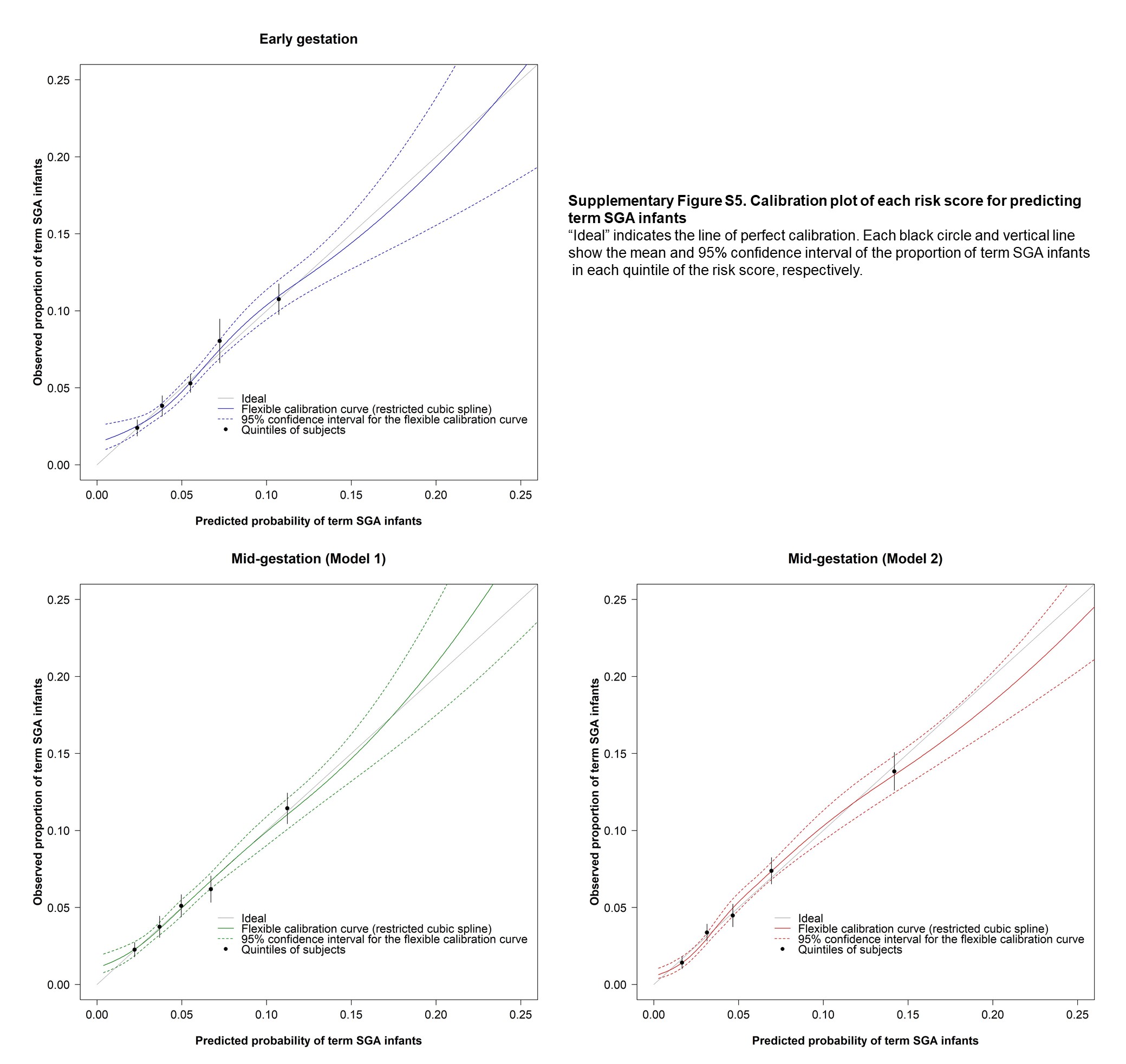

Supplement: Supplementary file 6 — Supplementary Information 6. [file 41598_2022_12892_MOESM6_ESM.jpg]

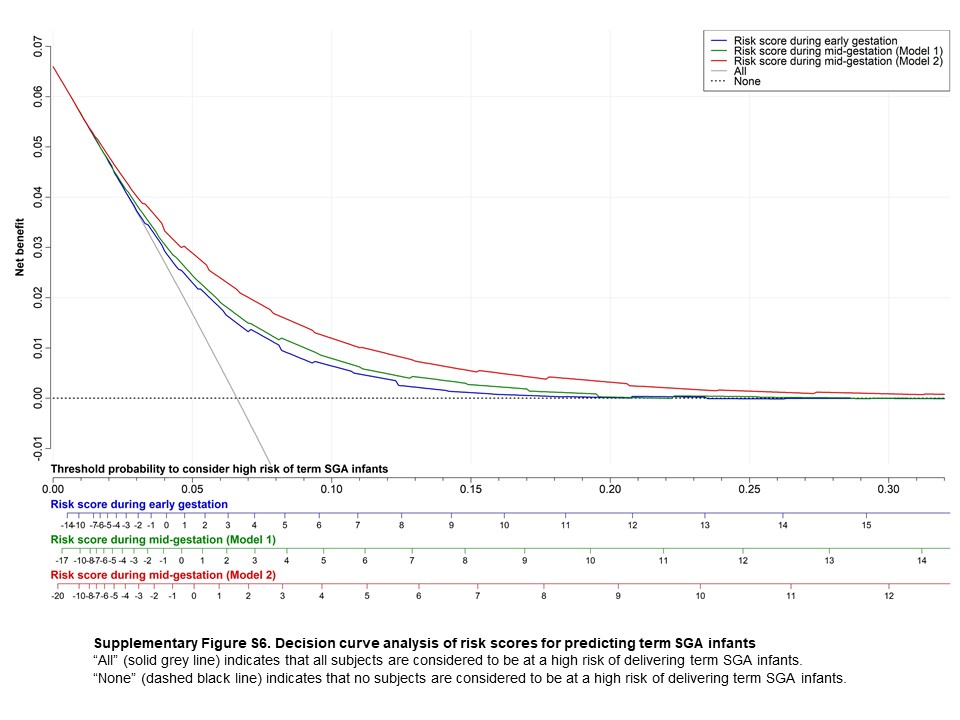

Supplement: Supplementary file 7 — Supplementary Information 7. [file 41598_2022_12892_MOESM7_ESM.jpg]
